# Synthesis, Structure and Cytoprotective Activity of New Derivatives of 4-Aryl-3-Aminopyridin-2(1*H*)-One

**DOI:** 10.3390/molecules30163331

**Published:** 2025-08-09

**Authors:** Zarina Shulgau, Irina Palamarchuk, Egor Dezhko, Shynggys Sergazy, Assel Urazbayeva, Yuliya Safarova, Alexander Gulyayev, Yuri Gatilov, Ivan Kulakov

**Affiliations:** 1 National Laboratory Astana, Nazarbayev University, Kabanbai Batyr Ave. 53, Astana Z05H0P9, Kazakhstan; shynggys.sergazy@gmail.com (S.S.); assel.urazbayeva@nu.edu.kz (A.U.); yantsen@nu.edu.kz (Y.S.); akin@mail.ru (A.G.); 2 CF “Institute of Innovative and Preventive Medicine”, Alikhan Bokeikhan Street, Building 1, Astana Z05M0M4, Kazakhstan; 3 Department of Science Development, Astana Medical University, Beibitshilik St 49/A, Astana Z10K9D9, Kazakhstan; 4 School of Natural Sciences, Tyumen State University, 15a Perekopskaya St., Tyumen 625003, Russia; i.v.palamarchuk@utmn.ru (I.P.);; 5 N.N. Vorozhtsov Novosibirsk Institute of Organic Chemistry, Siberian Branch of the Russian Academy of Science, 9 Akademika Lavrientieva Ave., Novosibirsk 630090, Russia; gatilov@nioch.nsc.ru

**Keywords:** 3-aminopyridin-2(1*H*)-one derivatives, 3-(arylmethyl)-6-methyl-4-arylpyridin-2(1*H*)-ones, Leuckart-Wallach and Eschweiler-Clarke reactions, X-ray structural analysis, cytoprotective activity

## Abstract

As a continuation of our research on the synthesis and study of biological properties of new derivatives of 3-aminopyridin-2(1*H*)-ones, we investigated the Leuckart–Wallach and Eschweiler–Clarke reactions with selected 3-aminopyridin-2(1*H*)-ones and 3-(arylmethyl)pyridin-2(1*H*)-ones. It was found that under the conditions of the Leuckart–Wallach reaction with aromatic aldehydes in formic acid, mainly formamides of the indicated 3-aminopyridones are formed. The Eschweiler–Clarke reaction of 3-aminopyridin-2(1*H*)-ones and 3-(arylmethyl)pyridin-2(1*H*)-ones with an aqueous solution of formaldehyde result in the formation of tertiary N–benzyl(methyl)amino)-pyridin-2(1*H*)-ones in almost quantitative yield. The 3-aminopyridin-2(1*H*)-ones derivatives synthesized by us were used for the biological screening of cytoprotective activity in the MTT (3-(4,5-dimethylthiazol-2-yl)-2,5-diphenyltetrazolium bromide) test to determine the viability of fibroblast cells isolated from the NIH/Swiss mouse embryo (NIH/3T3, Gibco). It was found that many of the studied compounds under the conditions of our experiment exhibited significant cytoprotective effects, thereby enhancing cell survival.

## 1. Introduction

One of the most pressing global issues in modern society, concerning all of humanity, is the problem of aging and the related challenges of age-associated diseases. These include cardiovascular, neurodegenerative, metabolic, oncological, and several other conditions. Aging is considered the main risk factor for many chronic diseases, which often emerge in old age [1]. Global demographic statistics from the past 20 years clearly indicate a steady increase in the number of elderly people in the population. According to WHO projections, by 2050, the number of people aged 60 and older will increase by 9% compared to 2014, from 12% (864 million, with a global population of 7.2 billion) to 21% (2.016 billion, with a global population of 9.6 billion). There is also growing concern about the increasing social burden on society associated with aging and its accompanying diseases [2,3].

This rising social burden explains the urgent need to develop methods for slowing the progression of age-associated diseases and to search for anti-aging drugs, including geroprotectors, which can help achieve a state of “healthy aging” [4,5]. However, as of today, there are either no effective geroprotectors in the pharmacological arsenal or very few. The lack of clinically proven geroprotective drugs is partly due to the limited pool of available biologically active substances that exhibit senolytic activity in vitro, stimulate autophagy in cellular aging models, and can prevent or slow the progression of age-related pathologies in vivo [6].

For many years, our research group has been engaged in the synthesis and biological activity studies of a relatively new class of compounds, 4-aryl(hetaryl)-substituted 3-aminopyridin-2(1*H*)-ones of type **1**, which are scarcely described in the literature. The methods of their synthesis, based on the intramolecular cyclization of N-(3-oxoalkenyl)amides, as well as the challenges related to their synthetic accessibility, have been described in detail in our work [7]. It is shown that almost all of the synthesized 3-aminopyridin-2(1*H*)-ones exhibit high antiradical activity. It is worth noting that antiradical activity is considered one of the general anti-aging mechanisms, beginning with the aging theory proposed by D. Harman (1956) and N.M. Emanuel (1958), and is being further developed in the present day [8,9].

**Figure 1:**
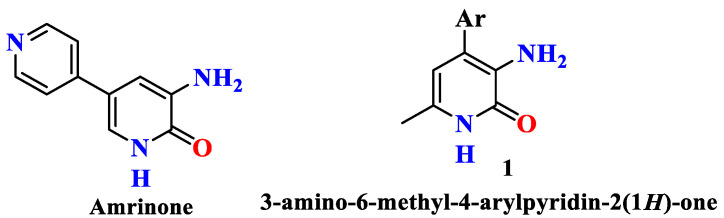
Structural formulas of Amrinone and 3-aminopyridin-2(1*H*)-one.

3-Aminopyridin-2(1*H*)-one derivatives have attracted significant interest due to their promising biological activity [10,11]. For example, the drug Amrinone (5-amino-[3,4′-bipyridin]-6(1*H*)-one) is an inhibitor of pyridine phosphodiesterase 3 and has a cardiotonic and vasodilating effect (Figure 1) [12]. Many other derivatives of 3-aminopyridin-2(1*H*)-one also exhibit antiviral activity, including against the AIDS virus [13,14]. The presence of an “integrated” amino acid fragment renders them attractive scaffolds for the synthesis of new derivatives with promising biological applications [15,16,17,18,19,20].

Our previous studies have demonstrated [21,22,23,24,25,26] that almost all derivatives of 3-aminopyridin-2(1*H*)-one **1** have high biological activity. Due to their strong biological activity, further exploration of novel derivatives is warranted.

In the course of preliminary studies, it was found that the primary amino group serves as an attractive reactive center for the further modification of the synthesized 4-aryl(hetaryl)-substituted 3-aminopyridin-2(1*H*)-ones. In the continuation of our studies, we also showed that reductive amination of 3-aminopyridin-2(1*H*)-one with aromatic aldehydes yielded 3-(arylmethyl)-6-methyl-4-phenylpyridin-2(1*H*)-ones, which also exhibited not only high antiradical and cytoprotective activity (these effects may be considered indicative of potential senolytic activity) [21,22] but also tranquilizing (anxiolytic) activity in the in vivo “dark-light chamber” test, as well as antidepressant activity in the “Porsolt passive swimming test” [23]. Several leaders were identified among the derivatives of 3-(arylmethylamino)-6-methyl-4-phenylpyridin-2(1*H*)-one with a higher potential neurotropic activity than the comparison drugs (mexidol and amitriptyline). It should be noted that the conjugates synthesized by us, on the basis of 3-aminopyridin-2(1*H*)-ones containing 1,3,4-thiadiazole ring and thiourea functional group, showed their high hypoglycemic and antidiabetic potential in inhibiting α-amylase and α-glucosidase [24,25,26], exceeding the effectiveness of the comparison drug acarbose.

Thus, the high pharmacological potential of 3-aminopyridin-2(1*H*)-one derivatives, confirmed by numerous examples, stimulates us to further search for new derivatives and study their biological activity. The development of methods for introducing new pharmacophoric groups into the 3-aminopyridone scaffold may hypothetically enhance biological activity and lead to the synthesis of new and original compounds with potential geroprotective properties.

## 2. Results and Discussion

### 2.1. Chemical Part

**Figure 2:**
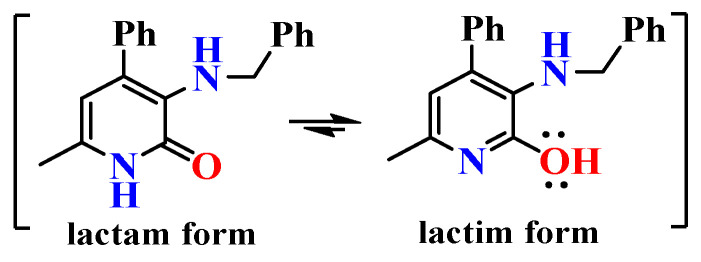
Tautomeric forms 3-(benzylamino)-6-methyl-4-phenylpyridin-2(1*H*)-one.

In order to find new possibilities for chemical modification of the structure of biologically active 3-(arylmethylamino)-4-phenylpyridin-2(1*H*)-ones **2** and to introduce another aromatic functional group into their structure, we carried out reactions with some aromatic aldehydes (benzaldehyde, 4-fluorobenzaldehyde, salicylic aldehyde) under various conditions (in a solution of polar and non-polar solvents, with the addition of acid catalysts, in a medium of phosphoric and formic acids). The main purpose of carrying out these reactions was to synthesize biologically relevant cyclic oxazolidines on the basis of 3-(arylmethylamino)-4-phenylpyridin-2(1*H*)-ones **2**. This cyclization reaction into oxazolidines is well applicable to the example of condensation of many 1,2-aminoalcohols and their derivatives with aromatic aldehydes [27]. And since it is known that lactim-lactam tautomerism and the associated binucleophilicity are possible for our starting compounds (Figure 2), we hypothesized that these compounds might exhibit properties analogous to those of 1,2-aminoalcohols.

For this purpose, we attempted to study the interaction of 3-(arylmethylamino)-4-phenylpyridin-2(1*H*)-one **1** with aromatic aldehydes under various conditions that could lead to cyclization into 1,2-dihydrooxazolo[5,4-b]pyridines **4**, which has not been described in the literature.

**Scheme 1:**
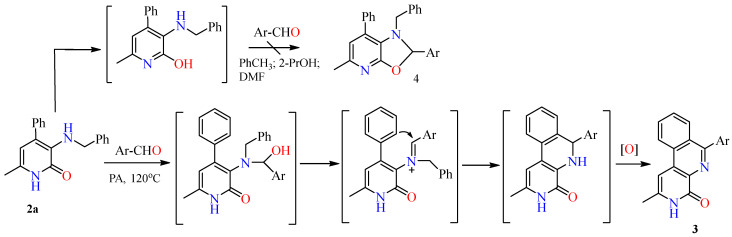
Reaction 3-(benzylamino)-6-methyl-4-phenylpyridin-2(1*H*)-one **2a** with aromatic aldehydes.

It has been shown that carrying out the reaction, for example, in a concentrated phosphoric (or polyphosphoric) acid at a temperature of 120–130 °C, leads mainly to the formation of the 6-arylbenzo[c][1,7]naphthyridine(3*H*)-ones **3** (Scheme 1) described by us earlier [28]. In this case, debenzylation occurs and under the conditions of the reaction with salicylic or thiophenecarbaldehyde, the elimination of phenol and thiophene also occurs, analogous with the examples that we described earlier in [28].

Attempts to prepare 1,2-dihydrooxazolo[5,4-b]pyridines of type **4** by heating the corresponding 3-aminopyridone **2a** with aromatic aldehydes under various conditions using polar protic and aprotic solvents, as well as non-polar solvents with both acidic and basic catalysts that shift the amide-imine tautomerism equilibrium, were unsuccessful.

**Figure 3:**
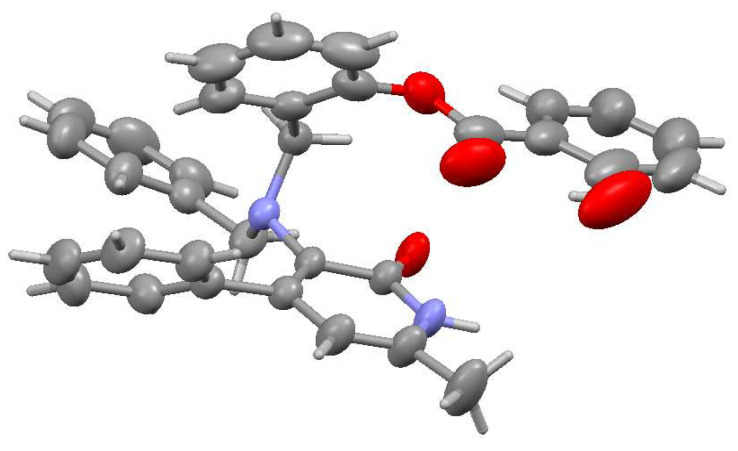
X-ray crystal structure of compound **6** (CCDC 2467189).

However, when carrying out the reaction of 3-[(benzyl)amino]pyridin-2(1*H*)-one **2a** with an excess of salicylic aldehyde in an alcohol medium in the presence of formic acid, we isolated a white crystalline substance with a good yield, the ^1^H and ^13^C NMR analysis of which showed the presence of two fragments from the salicylic aldehyde in the structure of compound **6** (Figure S1 Supporting Information). X-ray structural analysis of the crystals of compound **6** confirmed the structure of the obtained derivative (Figure 3). Analysis of the structure of compound **6** also showed that the general mechanism of the first stage of the reaction corresponds to the mechanism of the Leuckart–Wallach reaction-reductive amination of the carbonyl compound [29,30,31]. The Leuckart–Wallach reaction is a unique method for obtaining important pharmaceuticals by introducing new aryl and alkyl fragments into the structure of aromatic amines [32].

**Scheme 2:**
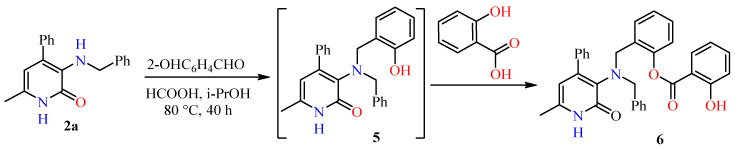
Reaction 3-(benzylamino)-6-methyl-4-phenylpyridin-2(1*H*)-one **2a** with excess salicylic aldehyde under Leuckart–Wallach reaction conditions.

Probably, the excess of salicylaldehyde used in the reaction under the influence of atmospheric oxygen led to oxidation of aldehyde into salicylic acid. Subsequent esterification of salicylic acid with intermediate phenol **5** resulted in target product **6** according to Scheme 2.

**Scheme 3:**
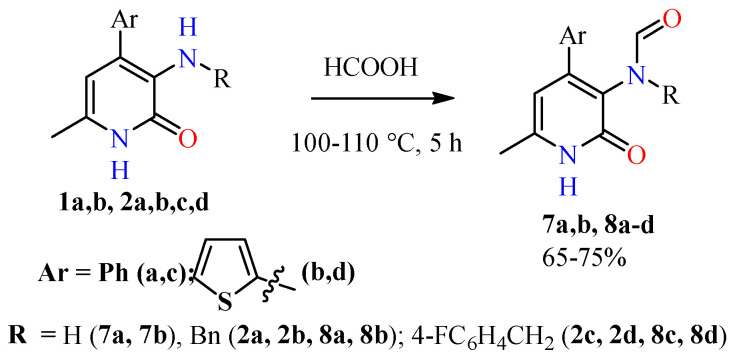
Synthesis of formamides **7a,b** and **8a**–**d**.

It was also found that 3-[(aryl)amino]pyridin-2(1*H*)-ones **2a,b** under the conditions of the Leuckart–Wallach reductive amination reaction in excess formic acid form the corresponding formamides **8a**–**d**. In this case, the reaction of 3-[(benzyl)amino]pyridin-2(1*H*)-ones **2a**–**d** with other aromatic aldehydes under similar reaction conditions led only to the formation of a small amount (depending on the amount of formic acid used) of formamides **8a**–**d** (Scheme 3). Formamides **7a,b** were also synthesized by boiling in formic acid with the corresponding 3-aminopyridones **1a,b**.

**Figure 4:**
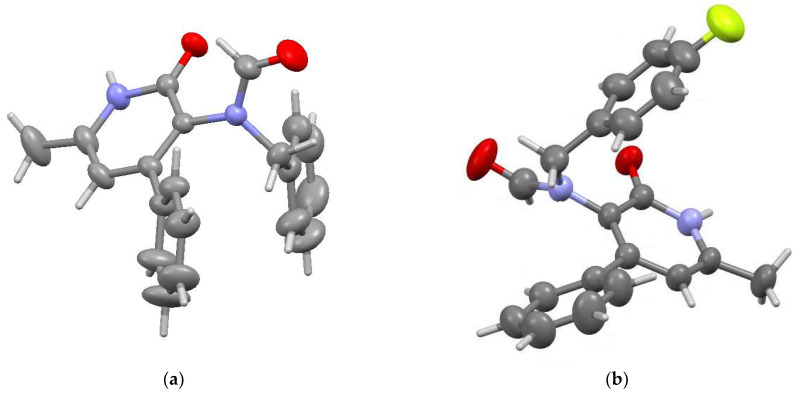
X-ray crystal structure of the compounds: (**a**) **8a**, CCDC 2467190; (**b**) **8c**, CCDC 2467191.

The structure of the obtained compounds was confirmed by ^1^H, ^13^C NMR spectroscopy (Figures S2–S7 Supporting Information) and additionally conducted X-ray diffraction analysis (Figure 4).

**Scheme 4:**
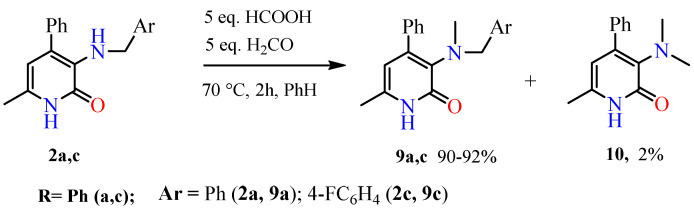
Reaction 4-phenylpyridin-2(1*H*)-ones **2a, c** with excess formaldehyde under Eschweiler–Clarke reaction conditions.

The most practical route involved a modification of the Leuckart–Wallach reaction (the Eschweiler–Clarke reaction [33,34]), as a result of which secondary 3-[(benzyl)amino]pyridin-2(1*H*)-one **2a** interacted with formaldehyde and formic acid to form *N*-methylated tertiary aminopyridones **9** (Scheme 4). Formaldehyde served as both the reagent and reducing agent. The reaction proceeded for about an hour with the release (precipitation) of the target reaction product from the solution in the form of a fine crystalline white powder.

Analysis of the reaction mixture by chromatograph mass spectrometry showed that the mixture contains two reaction products with retention times t_R_ = 10.81 and 17.79 min (Figure S13 Supporting Information).

**Figure 5:**
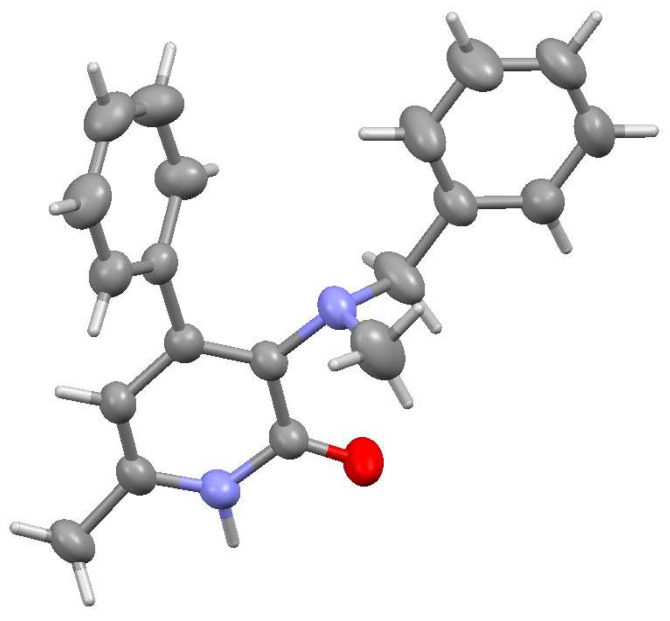
X-ray crystal structure of compound **9** (CCDC 2467192).

The reaction product with retention time t_R_ = 17.79 min and content of 95% has a molecular ion peak [M]^+^ = 304.16 with an intensity of 40%, which corresponds to the molecular mass of the tertiary amine **9a** (Figure S14 Supporting Information). The structure of this substance has been unambiguously confirmed by ^1^H NMR spectroscopy (Figure S8 Supporting Information) and additionally conducted X-ray structural analysis (Figure 5). In the ^1^H NMR spectrum of compound **9a**, the methylene protons of the arylmethyl fragment are equivalent and appear as a clear singlet, indicating the absence of interaction with the NH proton, which is recorded in the initial 3-(benzylamino)-6-methyl-4-phenylpyridin-2(1*H*)-one **2a** doublet.

**Scheme 5:**
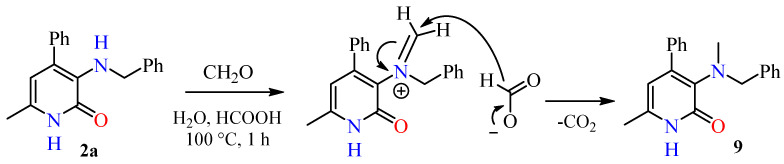
The mechanism of the methylation reaction of 3-(benzylamino)-6-methyl-4-phenylpyridin -2(1*H*)-one **2a** by the Eschweiler–Clarke reaction.

The formation of reaction products probably occurs through the stage of formation of an imine salt by the general mechanism of the Leuckart–Wallach reaction (Scheme 5), which then, depending on the reagent (formic acid or intramolecular cyclization), forms tertiary 3-(benzyl-(methyl)amino)pyridone **9.**

In addition, the reaction mixture contains 2% of a compound with a retention time of 10.81 min (Figure S13 Supporting Information). The peak of its molecular ion [M]^+^ is 228.13 (intensity 100%), and the peak of one of the fragments is 213.12 (74%) and 44.07 (28%), respectively (Figure S14 Supporting Information). Fragmentary analysis of the fragments suggests the formation of 3-(dimethylamino)-6-methyl-4-phenylpyridin-2(1*H*)-one **10** (Scheme 4).

**Scheme 6:**
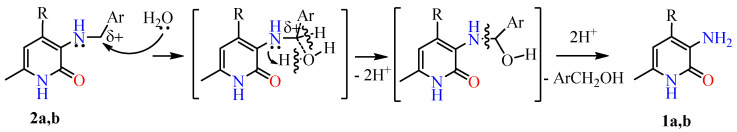
Proposed mechanism of the debenzylation side reaction of 3-(benzylamino)-6-methyl-4-phenylpyridin-2(1*H*)-one **2a** in the Eschweiler–Clarke reaction.

The formation of this product was unexpected. Probably, the minor formation of *N*,*N*-dimethylaminopyridone is associated with the elimination of the benzyl fragment under the reaction conditions and the subsequent methylation of the resulting methylaminopyridone (Scheme 6). A similar debenzylation process is described in the work [35].

**Scheme 7:**
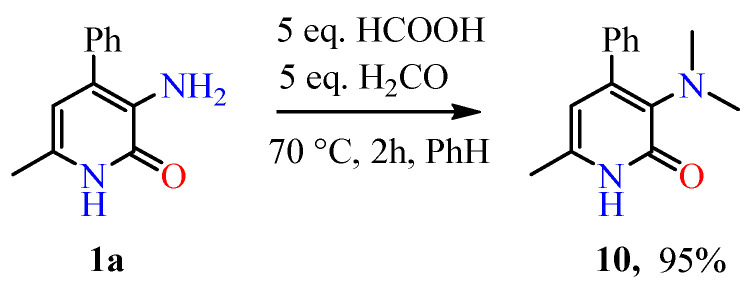
Reaction methylation 3-amino-6-methyl-4-phenylpyridin-2(1*H)*-one **1a** by the Eschweiler–Clarke reaction.

To prove the formation of *N*, *N*-dimethylaminopyridone, we performed a counter synthesis with the starting 3-aminopyridone **1a** under Eschweiler–Clark reaction conditions with excess formaldehyde and formic acid (Scheme 7). As a result, *N*,*N*-dimethylaminopyridone **10** was formed in almost quantitative yield (Figure S14 Supporting Information).

Attempts to optimize the reaction of formation of benzylmethylaminopyridone **9** led to the following results. Changing the solvent to a more polar one leads to an increase in the formation of product **9** by several percent. Changing the amount of formic acid, as well as increasing the excess of formalin, leads to a significant increase in the yield *N*,*N*-dimethylaminopyridone (up to 7%). Prolonged heating does not affect the yield of reaction products. Thus, to increase the yield of tertiary aminopyridone type **9**, it is necessary to use non-polar solvents and small excesses of formaldehyde and formic acid.

The Eschweiler–Clarke reaction was further investigated on 3-(4-fluorophenylmethylamino)-6-methyl-4-phenylpyridin-2(1*H*)-one **2** with similar results (Scheme 4, Figures S16 and S17 Supporting Information).

**Scheme 8:**
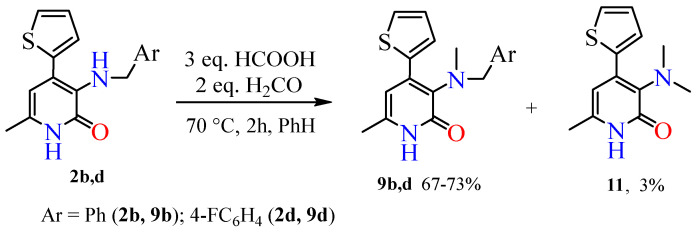
Reaction 3-(benzylamino)-6-methyl-4-(thiophen-2-yl)pyridin-2(1*H*)-one **2b** c in excess formaldehyde conditions by the Eschweiler–Clarke reaction.

In attempts to obtain new tertiary amines with a thiophene substituent in the pyridine ring, it was found that the Eschweiler–Clarke reaction must be carried out under milder conditions, since under harsh conditions, the secondary aminopyridone **2b,d** is destroyed and the tertiary amine **9b,d** released decreases (Scheme 8, Figures S18 and S19 Supporting Information).

**Scheme 9:**
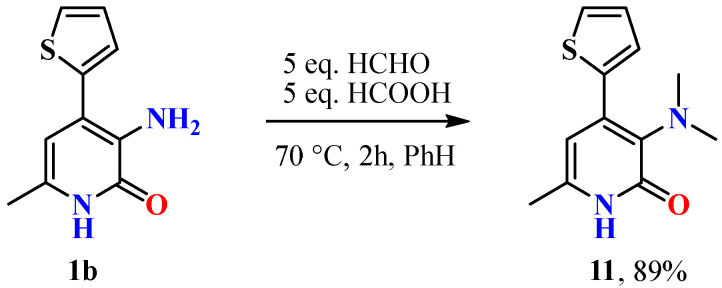
Methylation reaction of 3-amino-6-methyl-4-(thiophen-2-yl)pyridin-2(1*H*)-one **1b** by the Eschweiler–Clarke reaction.

Also, to prove the formation of *N*,*N*-dimethylpyridone **11**, a counter synthesis of the starting 3-aminopyridone **1b** was carried out under the conditions of the Eschweiler-Clarke reaction, with results similar to those in the previous reactions (Scheme 9).

Thus, the reaction carried out to obtain tertiary aminopyridones **9a**–**d**, **10** and **11**, which have not been described previously in the literature, may be of interest in terms of a convenient method for obtaining a new library of compounds for subsequent study of biological activity and possible study of the structure–activity relationship.

### 2.2. Structural Features of Compounds ***6***, ***8a***, ***8c***, ***9a***

In crystals of compounds **6**, **8a**, **8c**, **9a**, the geometry of the pyridine-2-one cycle is close to the geometry of this cycle in the compound 3-[(furan-2-ylmethyl)-amino]-6-methyl-4-phenyl-1*H*-pyridin-2-one [21]. The phenyl groups in the 4-position are inverted from the pyridine plane by 47.7 (6) (**6**)–55.2° (**9a**). Note the pyramidality of the amino nitrogen atom of compounds **6** and **9a** (the sums of the valence angles are 336.8° and 339.5°). The presence of a formamide fragment in compounds **8a** and **8c** leads to a flattening of the nitrogen atom (the sums of the valence angles are 359.5° and 359.6°). In these compounds, the angles between the NC_3_ and pyridine planes are the same: 59.2° and 59.4°. Dimers are formed in crystals of compounds **6**, **8a**, **8c**, **9a** due to NH∙∙∙O hydrogen bonds between pyridine-2-one cycles. The range of parameters of hydrogen bonds are as follows: distances H∙∙∙O 1.78(2)–1.93(3) Å, N∙∙∙O 2.722(2)–2.799(4) Å, angles NH∙∙∙O 167(3)–177(2)°.

### 2.3. Cytoprotective Activity Tests

The cytoprotective properties of the compounds **7a,b**, **8a**–**d**, **9a**–**d** were evaluated in the MTT (3-(4,5-dimethylthiazol-2-yl)-2,5-diphenyltetrazolium bromide) test on the fibroblast cell line that was isolated from a mouse NIH/Swiss embryo (NIH/3T3, Gibco). Compounds **7a,b**, **8a**–**d**, **9a**–**d** were studied at concentrations 25, 50, and 100 mM. Compounds **7a,b**, **8a**–**d**, **9a**–**d** were dissolved in 10% DMSO. Additionally, 10 μL of the dissolved substance was added to 100 μL of the nutrient medium with fibroblasts. Cells cultured in the medium supplemented with 10% DMSO served as controls. After cells were incubated for 24 h with test compounds, viability was determined in the MTT test using the MTT Cell Proliferation Assay Kit (BioVision) according to the manufacturer’s instructions.

Table 1 shows the average values of cell survival (in % of control) for three measurements.

Table 1 shows the cell viability indices of the NIH/3T3 culture after incubation with compounds **7a,b**, **8a**–**d**, **9a**–**d**. The change in cell viability under the influence of the studied 3-aminopyridin-2(1*H*)-one derivatives was expressed as a percentage relative to the viability of NIH/3T3 cell line, taken as 100% viability in the control (cells without the addition of test compounds).

## 3. Discussion

As can be seen, none of the tested compounds demonstrated cytotoxicity in the MTT assay, since cell survival in all cases was not significantly lower than the control, which was taken as 100% cell survival.

The values of the cell viability index in the presence of the studied compounds are, in most cases, significantly higher than the control. These findings suggest that the studied compounds **7a,b**, **8a**–**d**, **9a**–**d** may exhibit some stimulating effect on the viability of the NIH/3T3 cell culture, or the studied compounds of a number of 3-aminopyridin-2(1*H*)-one derivatives may counteract the spontaneous decline in cell viability in the culture, which naturally develops over time. The latter assumption, taking into account the results of previously performed work, seems more plausible, and we therefore interpret the observed effects as cytoprotective of the above-described 3-aminopyridin-2(1*H*)-one derivatives.

From the data presented in Table 1 it is evident that incubation of NIH/3T3 cells with compounds **7a,b**, **8a**–**d**, **9a**–**d** exhibited a marked, dose-dependent cytoprotective effect, likely preventing cell damage during the 24 h incubation period. Moreover, after 24 h, their viability significantly exceeds that of the control cells. A clearly dose-dependent cytoprotective response is observed for the following compounds: **7b**, **8a**, **8b**, **8d**, **9b**, **9c**.

Future studies should aim to expand the understanding of the potential cytoprotective effect of compounds **7b**, **8a**, **8b**, **8d**, **9b**, **9c**, especially by introducing substances/agents that damage cells (e.g., doxorubicin) into the incubation system of cultured cells. It is also necessary to expand the set of targets for compounds to use not only cell lines but also primary cell cultures.

At present, as a result of the study of the biological activity of new synthesized compounds, it is possible to state the probability of the presence of cytoprotective potential.

Notably, documented cytoprotective effects of synthetic compounds are relatively rare and is mainly described as the effect of plant compounds [36,37,38,39,40].

The identification of a hypothetical cytoprotective effect in a series of compounds predetermines interest in the study of these compounds, **7b**, **8a**, **8b**, **8d**, **9b**, and **9c**, in pharmacology, toxicology, and also in anti-aging studies.

## 4. Materials and Methods

The description of this section (figures of spectrums) is included as Supplementary Material.

^1^H and ^13^C NMR spectra were recorded on a Bruker DRX400 («Bruker BioSpin GmbH», Ettlingen, Germany) (400 and 100 MHz, respectively), Bruker AVANCE 500 («Bruker BioSpin GmbH», Germany) (500 and 125 MHz, respectively), and Magritek spinsolve 80 carbon ultra (Aachen, Germany) (81 and 20 MHz, respectively) instruments using DMSO-d_6_ or CDCl_3_. The internal standard was residual solvent signals (7.25 and 77.0 ppm ^1^H and for ^13^C nuclei in CDCl_3_ and 2.49 and 39.9 ppm ^1^H and for ^13^C nuclei in DMSO-d_6_). Elemental analysis was performed with a Carlo Erba 1106 CHN analyzer (Milan, Italy). Melting points were determined using a Stuart SMP10 (Stuart, UK) hot bench. Monitoring of the reaction course and the purity of the products was carried out by TLC (Merck, Darmstadt, Germany) on Sorbfil plates and visualized using iodine vapor or UV light.

X-ray diffraction data for compounds **6**, **8a**, **9a** were obtained at room temperature with a Bruker Kappa Apex II CCD diffractometer (Bruker, Germany) with Mo-Kα radiation (λ = 0.71073 Å) and a graphite monochromator using ϕ, ω scans of narrow frames. Experimental data reduction was performed using APEX2 v2012.2-0 suite. Absorption corrections were applied empirically using the SADABS-2008/1 programs. X-ray diffraction data for compound **8c** were obtained at room temperature with a Xcalibur Ruby CCD diffractometer (Oxford, UK) with Cu-Kα radiation (λ = 1.54184 Å) and a graphite monochromator using ω scans. Experimental data reduction and absorption corrections were performed using CrysAlis171 suite (version 1.171.38.43d). The structures were solved by direct methods and refined by the full-matrix least-squares method against all F^2^ in the anisotropic approximation using the SHELX 2018 set of programs. The positions of HN hydrogen atoms were located from the difference map and refined isotropically. Positions of the rest of the H atoms were refined with the riding model. CCDC 2467189-2467192 contain the supplementary crystallographic data for this paper. These data can be obtained free of charge from The Cambridge Crystallographic Data Center https://www.ccdc.cam.ac.uk/structures/ (deposited on 25 June 2025).

Chromato-mass spectrometric studies were carried out on a Trace GC Ultra chromatograph (Thermo Fisher Scientific Inc., Waltham, MA, USA) with a DSQ II mass-selective detector in the electron ionization mode (70 eV) on a Thermo TR-5 MS quartz capillary column, 15 m long, 0.25 mm inner diameter, with a film thickness of the stationary phase of 0.25 μm. Splitless input mode was used. The carrier gas discharge was 20 mL/min. The velocity of the carrier gas (helium) was 1 mL/min. The evaporator temperature was 200 °C, transition chamber temperature was 200 °C, and ion source temperature was 200 °C. The temperature of the column thermostat was changed according to the program from 15 (5 min delay) to 220 °C at a rate of 20 °C per min, to 290° at a rate of 15° per min. The total analysis time was 30 min. The volume of the injected sample is 1 μL. Chromatograms were recorded in TIC mode. The range of mass scanning is 30–450 amu.

### 4.1. X-Ray Structural Study of Product

Crystal data **6**: C_33_H_28_N_2_O_4_, M = 516.57, monoclinic, space group *C* 2/*c*, at 296 K: *a* = 17.4826(8), *b* = 13.5157(7), *c* = 24.1512(9) Å, β = 97.384(2)°, *V* = 5659.4(4) Å^3^, *Z* = 8, *d*_calc_ = 1.213 g∙cm^−3^, μ = 0.080 mm^−1^, a total of 49,730 (θ_max_ = 26.05°), 5593 unique (*R*_int_ = 0.0533), 3758 [*I* > 2σ(*I*)], 357 parameters. GooF = 0.960, *R*_1_ = 0.0518, *wR*_2_ = 0.1406 [*I* > 2σ(*I*)], *R*_1_ = 0.0857, *wR*_2_ = 0.1740 (all data), max/min diff. peak 0.235/−0.178 e·Å^−3^. CCDC 2467189.

Crystal data **8a**: C_20_H_18_N_2_O_2_, M = 318.36, triclinic, space group *P* −1, at 296 K: *a* = 7.7481(3), *b* = 8.9662(4), *c* = 13.3817(6) Å, α = 98.494(2), β = 04.602(2), γ = 103.312(2)°, *V* = 854.29(6) Å^3^, *Z* = 2, *d*_calc_ = 1.238 g·cm^−3^, μ = 0.081 mm^−1^, a total of 29,182 (θ_max_ = 30.09°), 5007 unique (R_int_ = 0.0398), 3344 [*I* > 2σ(*I*)], 222 parameters. GooF = 1.016, *R*_1_ = 0.0528, *wR*_2_ = 0.1391 [*I* > 2σ(*I*)], *R*_1_ = 0.0867, *wR*_2_ = 0.1709 (all data), max/min diff. peak 0.268/−0.192 e·Å^−3^. CCDC 2467190.

Crystal data **8c**: C_20_H_17_FN_2_O_2_, M = 336.35, triclinic, space group *P* −1, at 296 K: *a* = 7.8797(8), *b* = 8.9057(10), *c* = 13.405(2) Å, α = 97.170(11), β = 106.466(11), γ = 103.214(9)°, *V* = 859.9(2) Å^3^, *Z* = 2, *d*_calc_ = 1.299 g∙cm^−3^, μ = 0.758 mm^−1^, a total of 5604 (θ_max_ = 75.99°), 3430 unique (*R*_int_ = 0.0602), 1479 [*I* > 2σ(*I*)], 231 parameters. GooF = 0.932, *R*_1_ = 0.0600, *wR*_2_ = 0.1090 [*I* > 2σ(*I*)], *R*_1_ = 0.1503, *wR*_2_ = 0.1493 (all data), max/min diff. peak 0.165/−0.217 e Å^−3^. CCDC 2467191.

Crystal data **9a**: C_20_H_20_N_2_O, M = 304.38, monoclinic, space group *P* 2_1_/*n*, at 296 K: *a* = 10.7039(4), *b* = 7.8392(3), *c* = 21.0351(8) Å, β = 102.114(2)°, *V* = 1725.75(11) Å^3^, *Z* = 4, *d*_calc_ = 1.172 g∙cm^−3^, μ = 0.073 mm^−1^, a total of 31,334 (θ_max_ = 27.94°), 4137 unique (*R*_int_ = 0.0508), 3160 [*I* > 2σ(*I*)], 214 parameters. GooF = 1.006, *R*_1_ = 0.0555, *wR*_2_ = 0.1473 [*I* > 2σ(*I*)], *R*_1_ = 0.0757, *wR*_2_ = 0.1736 (all data), max/min diff. peak 0.214/−0.206 e·Å^−3^. CCDC 2467192.

### 4.2. Synthesis of Compounds

#### 4.2.1. 2-((Benzyl(6-methyl-2-oxo-4-phenyl-1,2-dihydropyridin-3-yl)amino)methyl)phenyl 2-Hydroxybenzoate **6**

To 1 mmol of 3-(benzylamino)-6-methyl-4-phenylpyridin-2(1H)-one 2a were added 5 mmol of salicylic and 5 mmol of formic acid. The reaction mixture was boiled in 10 mL of isopropyl alcohol for 24 h and then poured onto ice. The resulting precipitate was filtered off, washed with water, and recrystallized from 2-propanol.

Yield: 219 mg (71%), yellow crystals, Mp: 161–162 °C. ^1^H NMR (400 MHz, CDCl_3_) δ ppm (*J*, Hz): 2.13 (s, 3H, CH_3_); 4.16 (s, 2H, N-CH_2_); 4.23 (s, 2H, N-CH_2_); 5.77 (s, 1H, H-5); 6.80 (d, *J* = 7.6, 2H, H-2.6 Bn); 6.85 (t, *J* = 7.6, 1H, H-5′Ar); 6.93 (d, *J* = 9.2, 1H, H-3′′ Ar); 7.02 (d, *J* = 7.6, 1H, H-6′ Ar); 7.09 (t, *J* = 7.6, 1H, H-4′ Ar); 7.13–7.17 (m, 6H, H-2,3,4,5,6 Ph, H-5′′ Ar); 7.18–7.24 (m, 3H, H-3,4,5 Bn); 7.26 (d, *J* = 7.6, 1H, H-3′ Ar); 7.40 (t, *J* = 6.1, 1H, H-4′′ Ar); 7.87 (d, *J* = 6.1, 1H, H-6′′ Ar); 10.42 (s, 1H, OH), 12.91 (br. s, 1H, NHCO); ^13^C NMR (100 MHz, CDCl_3_) δ ppm: 18.2 (CH_3_); 49.1 (N-CH_2_); 56.8 (N-CH_2_); 108.5 (C-5); 111.7; 117.6; 119.2; 121.6; 126.2; 126.8; 127.3; 127.6 (2C Ph); 127.7; 128.0 (2C Ph); 128.3 (2C Ph); 129.1 (2C Ph); 130.4; 131.6; 131.8; 133.1; 136.1; 139.1; 139.2; 140.8; 148.5; 150.8; 162.0; 164.4; 168.7. Found: C, 76.85; H, 5.57; N, 5.38. Calculated for C_33_H_28_N_2_O_4_: C, 76.73; H, 5.46; N, 5.42.

#### 4.2.2. The General Procedure for Obtaining Formamide Derivatives **7a**,**b**, **8a**–**d**

To 1.0 mmol of the corresponding 3-aminopyridone derivatives **1a,b**; **2a**–**d** was added 50.0 mmol of formic acid. The reaction mixture was heated for 2 h and then poured onto ice. The aqueous layer was extracted with ethyl acetate (3 × 25 mL), the organic layer was dried over Na_2_SO_4_, and the solvent was distilled off under reduced pressure. The products were recrystallized from 2-propanol-hexane (1:2).

#### 4.2.3. *N*-(6-Methyl-2-oxo-4-phenyl-1,2-dihydropyridin-3-yl)formamide **7a**

Yield: 151 mg (66%), colorless crystals, Mp: 210–211 °C. ^1^H NMR (81 MHz, DMSO-d_6_) δ ppm (*J*, Hz): 2.20 (s, 3H, CH_3_); 6.00 (s, 1H, H-5); 7.38 (br.s, 5H, H-2,3,5,6 Ph); 7.95 (s, 1H, CHO); 8.78 (br.s, 1H, NHCO); 11.91 (s, 1H, NHCO). ^13^C NMR (20 MHz, DMSO-d_6_) δ ppm: 18.3 (CH_3_); 106.0 (C-5); 120.0; 127.6; 128.3 (4C Ph); 137.0; 137.8; 143.0; 160.5; 164.9. Found: C, 68.19; H, 5.53; N, 12.02. Calculated for C_13_H_12_N_2_O_2_: C, 68.41; H, 5.30; N, 12.27.

#### 4.2.4. *N*-(6-Methyl-2-oxo-4-(thiophen-2-yl)-1,2-dihydropyridin-3-yl)formamide **7b**

Yield: 152 mg (65%), colorless crystals, Mp: 240–241 °C. ^1^H NMR (81 MHz, DMSO-d_6_) δ ppm (*J*, Hz): 2.20 (s, 3H, CH_3_); 6.39 (s, 1H, H-5); 7.14 (br.s, 1H, H-3 thiophene); 7.66–7.72 (br.m, 2H, H-4, 5 thiophene); 8.10 (br.d, *J* = 13.5, 1H, CHO); 9.30 (br.s, 1H, NHCO’); 11.79 (br.s, 1H, NHCO). ^13^C NMR (20 MHz, DMSO-d_6_) δ ppm: 18.4 (CH_3_); 103.2 (C-5); 127.4; 128.9; 129.4; 130.1; 132.2; 142.9; 160.6; 161.6; 165.6. Found: C, 56.06; H, 5.68; N, 11.66. Calculated for C_11_H_10_N_2_O_2_S: C, 56.40; H, 4.30; N, 11.96.

#### 4.2.5. *N*-Benzyl-*N*-(6-methyl-2-oxo-4-phenyl-1,2-dihydropyridin-3-yl)formamide **8a**

Yield: 152 mg (65%), white powder, Mp: 2 40–241 °C. ^1^H NMR (81 MHz, DMSO-d_6_) δ ppm (*J*, Hz): 2.17 (s, 3H, CH_3_); 4.34 (br.s, 2H, N-CH_2_); 5.95 (s, 1H, H-5); 6.86–7.37 (m, 10H, H-2,3,4,5,6 Ph, H-2′, 3′, 4′, 5′, 6′ Ph); 8.10 (s, 1H, CHO); 12.02 (s, 1H, NHCO). ^13^C NMR (20 MHz, DMSO-d_6_) δ ppm 18.3 (CH_3_); 46.3 (N-CH_2_); 106.3 (C-5); 123.6; 127.1 (C Ph); 127.5 (2C Ph); 128.0 (3C Ph); 128.5 (4C Ph); 136.3; 136.8; 144.5; 149.6; 161.1; 164.4. Found: C, 75.16; H, 5.42; N, 9.03. Calculated for C_20_H_18_N_2_O_2_: C, 75.45; H, 5.70; N, 8.80.

#### 4.2.6. *N*-Benzyl-*N*-(6-methyl-2-oxo-4-(thiophen-2-yl)-1,2-dihydropyridin-3-yl)formamide **8b**

Yield: 211 mg (65%), light yellow crystals, Mp: 230–231 °C. ^1^H NMR (81 MHz, DMSO-d_6_) δ ppm (*J*, Hz): 2.17 (s, 3H, CH_3_); 4.18 (d, *J* = 14.1, 1H, N-CH_a_); 4.79 (d, *J* = 14.2, 1H, N-CH_b_); 6.29 (s, 1H, H-5); 7.11 (br.s, 6H, H-4 thiophene, H-2,3,4,5,6 Ph); 7.35 (d, *J* = 2.7, 1H, H-5 thiophene); 7.74 (d, *J* = 5.0, 1H, H-3 thiophene); 8.07 (s, 1H, CHO); 11.89 (s, 1H, NHCO). ^13^C NMR (20 MHz, DMSO-d_6_) δ ppm: 18.4 (CH_3_); 47.4 (N-CH_2_); 104.1 (C-5); 121.9; 127.2; 127.5; 127.8 (2C Ph); 129.0 (2C Ph); 129.3; 130.1; 135.8; 136.4; 142.2; 144.6; 161.3; 164.6. Found: C, 66.98; H, 4.56; N, 9.00. Calculated for C_18_H_16_N_2_O_2_S: C, 66.65; H, 4.97; N, 8.64.

#### 4.2.7. *N*-(4-Fluorobenzyl)-*N*-(6-methyl-2-oxo-4-phenyl-1,2-dihydropyridin-3-yl)formamide **8c**

Yield: 261 mg(76%), light brown crystals, Mp: 234–235 °C. ^1^H NMR (81 MHz, DMSO-d_6_) δ ppm (*J*, Hz): 2.18 (s, 3H, CH_3_); 4.20 (br.d, *J* = 15.4, 2H, N-CH_2_); 5.97 (s, 1H, H-5); 6.77–7.03 (m, 6H, H-3.5′ Ar, H-2.3,5.6 Ph); 7.30–7.35 (m, 3H, H-2.6′ Ar, H-4 Ph); 8.11 (s, 1H, CHO); 12.03 (s, 1H, NHCO). ^13^C NMR (20 MHz, DMSO-d_6_) δ ppm: 18.3 (CH_3_); 45.8 (N-CH_2_); 106.2 (C-5); 114.8 (d, ^2^*J*_13C-F_ = 21.2, C-3′,5′Ar); 123.5; 126.9; 127.5 (C-3;5 Ph); 127.9; 128.4 (C-2,6 Ph); 130.5 (d, ^3^*J*_13C-F_ = 8.1, C-2′,6′ Ar); 132.6; 136.7; 144.7; 149.9; 161.1 (d, ^1^*J*_13C-F_ = 225.8, C-4′ Ar); 164.5. Found: C, 71.65; H, 5.48; N, 8.51. Calculated for C_20_H_17_FN_2_O_2_: C, 71.42; H, 5.09; N, 8.33.

#### 4.2.8. *N*-(4-Fluorobenzyl)-*N*-(6-methyl-2-oxo-4-(thiophen-2-yl)-1,2-dihydropyridin-3-yl)formamide **8d**

Yield: 252 mg (73%), colorless powder, Mp: 234–235 °C. ^1^H NMR (81 MHz, DMSO-d_6_) δ ppm (*J*, Hz): 2.18 (s, 3H, CH_3_); 4.08–4.84 (m, 2H, N-CH_2_); 6.32 (s, 1H, H-5); 6.83–7.13 (br.m, 5H, H-4 thiophene, H-2,3,5,6 Ph); 7.39 (br.s, 1H, H-5 thiophene); 7.72 (br.s, 1H, H-3 thiophene); 8.06 (s, 1H, CHO); 11.90 (s, 1H, NHCO). ^13^C NMR (20 MHz, DMSO-d_6_) δ ppm: 18.4 (CH_3_); 46.7 (N-CH_2_); 104.1 (C-5); 114.5 (d., ^2^*J*_13C-F_ = 21.3, 2C, C-3′,5′ Ar), 121.8; 127.5; 129.4; 130.2; 131.1 (d., ^3^*J*_13C-F_ = 8.3, 2C, C-2′,6′ Ar), 132.0; 132.2; 136.3; 142.4; 144.8; 161.4; 164.7 (d, ^1^*J*_13C-F_ = 188.7, C-4′ Ar). Found: C, 63.55; H, 4.23; N, 9.49. Calculated for C_18_H_15_FN_2_O_2_S: C, 63.14; H, 4.42; N, 8.18.

#### 4.2.9. The General Procedure for the Preparation of 3-(Aryl(methyl)amino)-pyridin-2(1*H*)-ones 9a-d and 3-Dimethylaminopyridin-2(1H)-ones **10**, **11**

To 1.0 mmol of 3-(arylmethylamino)pyridin-2(1*H*)-one **2a**–**d** (for compounds **9a**–**d**) or 3-aminopyridin-2(1*H*)-one **1a,b** (for compounds **10,11**) was added a solution of 2.0 mmol of 40% formaldehyde and 3.0 mmol of formic acid. The reaction mixture was heated in 10 mL of benzene for 2 h, then poured onto ice. The aqueous layer was extracted with ethyl acetate (3 × 25 mL), the organic layer was dried over Na_2_SO_4_, and the solvent was distilled off on a rotary evaporator. The products were recrystallized from a mixture of 2-propanol and hexane (1:2).

#### 4.2.10. 3-(Benzyl(methyl)amino)-6-methyl-4-phenylpyridin-2(1*H*)-one **9a**

Yield: 279 mg (92%), colorless crystals, Mp: 146–147 °C. Mass spectrum (EI, 70 eV), *m*/*z* (*I* rel (%)): 304 [M]^+^ (52); 289 (70); 213 (100); 91 (30). ^1^H NMR (500 MHz, CDCl_3_) δ ppm (*J*, Hz): 2.37 (s, 3H, CH_3_); 2.60 (s, 3H, CH_3_); 4.13 (s, 2H, N-CH_2_); 5.97 (s, 1H, H-5); 7.06 (dd, ^3^*J* = 1.7, ^4^*J* = 1.7, 2H, H-2′,6′ Ar); 7.15–7.17 (m, 3H, H-3′, 4′, 5′ Ar); 7.24–7.26 (m, 2H, H-2,6 Ph); 7.35–7.40 (m, 3H, H-3,4,5 Ph); 13.05 (s, 1H, NHCO). ^13^C NMR (125 MHz, CDCl_3_) δ ppm: 18.5 (CH_3_); 40.1 (CH_3_); 59.0 (N-CH_2_); 108.5 (C-5); 126.5; 127.5; 127.80 (2C Ph); 127.83 (2C Ph); 128.6 (2C Ph); 128.7 (2C Ph); 135.7; 139.4; 139.7; 140.1; 149.1; 164.8. Found: C, 79.02; H, 6.77; N, 9.38. Calculated for C_20_H_20_N_2_O: C, 78.92; H, 6.62; N, 9.20.

#### 4.2.11. 3-(Benzyl(methyl)amino)-6-methyl-4-(thiophen-2-yl)pyridin-2(1*H*)-one **9b**

Yield: 226 mg (73%), yellow crystals, Mp: 178–179 °C. Mass spectrum (EI, 70 eV), *m*/*z* (*I* rel (%)): 310 [M]^+^ (28); 295(14); 219 (100); 91 (42). ^1^H NMR (81 MHz, DMSO-d_6_) δ ppm (*J*, Hz): 2.09 (s, 3H, CH_3_); 2.60 (s, 3H, CH_3_); 4.25 (s, 2H, N-CH_2_); 6.37 (s, 1H, H-5); 7.14 (br.s, 6H, H-4 thiophene, H-2,3,4,5,6 Ph); 7.60–7.64 (br.m, 2H, H-3,5 thiophene); 11.36 (s, 1H, NHCO). ^13^C NMR (20 MHz, DMSO-d_6_) δ ppm: 18.3 (CH_3_); 39.9 (CH_3_); 57.5 (N-CH_2_); 101.6 (C-5); 126.0 (1C thiophene); 126.8 (1C thiophene); 127.6 (2C Ph); 128.0 (1C Ph); 129.7 (2C Ph); 131.2 (1C thiophene); 131.6; 136.6; 138.2; 140.4; 141.2; 162.1. Found: C, 68.85; H, 5.98; N, 9.24. Calculated for C_18_H_18_N_2_OS: C, 69.65; H, 5.85; N, 9.02.

#### 4.2.12. 3-((4-Fluorobenzyl)(methyl)amino)-6-methyl-4-phenylpyridin-2(1*H*)-one **9c**

Yield: 289 mg (90%), light yellow crystals, Mp: 121–122 °C. Mass spectrum (EI, 70 eV), *m*/*z* (*I* rel (%)): 322 [M]^+^ (44); 307(46); 213 (100); 109 (54). ^1^H NMR (100 MHz, CDCl_3_) δ ppm (*J*, Hz): 2.36 (s, 3H, CH_3_); 2.61 (s, 3H, CH_3_); 4.06 (s, 2H, N-CH_2_); 5.97 (s, 1H, H-5); 6.82 (t, *J* = 8.8, 2H, H-3′, 5′ Ar); 6.98 (dd, 2H, H-2′, 6′ Ar); 7.24–7.26 (m, 2H, H-2.6 Ph); 7.36–7.39 (m, 3H, H-3,4,5 Ph); 13.01 (s, 1H, NHCO). ^13^C NMR (100 MHz, CDCl_3_) δ ppm: 18.5 (CH_3_); 40.1 (CH_3_); 58.3 (N-CH_2_); 108.5 (C-5); 114.5 (d, ^2^*J*_13C-F_ = 20.8, C-3′,5′ Ar), 127.6; 127.8 (2C Ph); 128.5 (2C Ph); 130.1 (d, ^3^*J*_13C-F_ = 7.7, C-2′,6′ Ar); 135.3; 135.4; 139.3; 140.3; 149.5; 160.7 (d, ^1^*J*_13C-F_ = 244.3, C-4′ Ar); 164.8. Found: C, 74.60; H, 6.08; N, 8.78. Calculated for C_20_H_19_FN_2_O: C, 74.51; H, 5.94; N, 8.69.

#### 4.2.13. 3-((4-Fluorobenzyl)(methyl)amino)-6-methyl-4-(thiophen-2-yl)pyridin-2(1*H*)-one **9d**

Yield: 219 mg (67%), yellow crystals, Mp: 189–190 °C. Mass spectrum (EI, 70 eV), *m*/*z* (*I* rel (%)): 328 [M]^+^ (36); 313(14); 219 (100); 109 (42). ^1^H NMR (81 MHz, DMSO-d_6_) δ ppm (*J*, Hz): 2.10 (s, 3H, CH_3_); 2.60 (s, 3H, CH_3_); 4.24 (s, 2H, N-CH_2_); 6.38 (s, 1H, H-5); 6.82–7.04 (m, 3H, H-4 thiophene, H-3,5 Ar); 7.19–7.36 (m, 2H, H-2,6 Ar); 7.60–7.65 (m, 2H, H-3,5 thiophene); 11.36 (s, 1H, NHCO). ^13^C NMR (20 MHz, DMSO-d_6_) δ ppm: 18.3 (CH_3_); 39.9 (CH_3_); 56.6 (-CH_2_); 101.7 (C-5); 114.3 (d, ^2^*J*_13C-F_ = 21.0, C-3′,5′ Ar); 126.0; 128.1; 130.8; 131.5; (d, ^3^*J*_13C-F_ = 8.0, C-2′,6′ Ar); 134.3; 134.5; 136.6; 140.5; 141.33; 161.2 (d, ^1^*J*_13C-F_ = 242.7, C-4′Ar); 162.2. Found: C, 65.98; H, 5.37; N, 8.68. Calculated for C_18_H_17_FN_2_ OS: C, 65.83; H, 5.22; N, 8.53.

#### 4.2.14. 3-(Dimethylamino)-6-methyl-4-phenylpyridin-2(1*H*)-one **10**

Yield: 217 mg (95%), yellow crystals, Mp: 148–149 °C. Mass spectrum (EI, 70 eV), *m*/*z* (*I* rel (%)): 228 [M]^+^ (100), 213 (66). NMR ^1^H (81 MHz, DMSO-d_6_) δ ppm (*J*, Hz): 2.11 (s, 3H, CH_3_); 2.49 (s, 6H, N(CH_3_)_2_); 5.81 (s, 1H, H-5); 7.33 (br.s, 5H, H-2,3,4,5,6 Ph); 11.55 (br.s, 1H, NHCO); NMR ^13^C (20 MHz, DMSO-d_6_) δ ppm: 18.1 (CH_3_); 42.4 (N(CH_3_)_2_); 106.2 (C-5); 127.4; 127.9 (2C Ph); 128.1 (2C Ph); 135.5; 139.3; 139.6; 145.8; 161.9. Found: C, 73.90; H, 7.27; N, 12.05. Calculated for C_14_H_16_N_2_O: C, 73.66; H, 7.06; N, 12.27.

#### 4.2.15. 3-(Dimethylamino)-6-methyl-4-(thiophen-2-yl)pyridin-2(1*H*)-one **11**

Yield: 208 mg (89%), yellow crystals, Mp: 198–199 °C. Mass spectrum (EI, 70 eV), *m*/*z* (*I* rel (%)): 234 [M]^+^ (100), 219 (44). Found: C, 61.70; H, 6.18; N, 11.79. Calculated for C_12_H_14_N_2_OS: C, 61.51; H, 6.02; N, 11.96.

### 4.3. Data Evaluation

Statistical processing of the results was carried out using the Excel program. The results obtained are presented as “mean ± standard error of the mean”.

## 5. Conclusions

Thus, we have investigated the Leuckart–Wallach and Eschweiler–Clarke reactions with selected 3-aminopyridin-2(1*H*)-ones and 3-(arylmethyl)pyridin-2(1*H*)-ones. It has been established that under the conditions of the Leuckart–Wallach reaction with aromatic aldehydes in formic acid, formamides of the indicated 3-aminopyridones are predominantly formed. The Eschweiler–Clarke reaction of 3-aminopyridin-2(1*H*)-ones and 3-(arylmethyl)pyridin-2(1*H*)-ones with an aqueous solution of formaldehyde leads to the formation of tertiary N-benzyl(methyl)amino)pyridin-2(1*H*)-ones in a virtually quantitative yield. The 3-aminopyridin-2(1*H*)-one derivatives synthesized by us were used for biological screening of cytoprotective activity in the MTT test (3-(4,5-dimethylthiazol-2-yl)-2,5-diphenyltetrazolium bromide) to determine the viability of fibroblast cells isolated from the NIH/Swiss mouse embryo (NIH/3T3, Gibco). It was found that many of the studied compounds exhibit a pronounced cytoprotective effect under the conditions of our experiment, thereby increasing cell survival.

## Figures and Tables

**Table 1:** The level of viability of NIH/3T3 cells under in vitro conditions under the influence of the studied samples in the MTT test.

No.	Compound		Sample Concentration, mM	The Average Values of Cell Survival (in % of Control)
1	**7a**	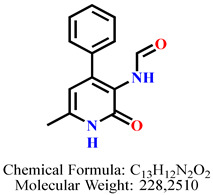	100 mM	118.0 ± 3.5
			50 mM	106.8 ± 4.3
			25 mM	100.5 ± 1.6
2	**7b**	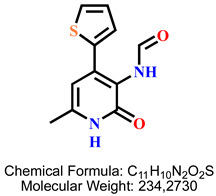	100 mM	1308.6 ± 114.1
			50 mM	764.9 ± 70.7
			25 mM	378.4 ± 24.4
3	**8a**	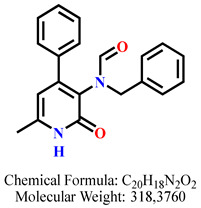	100 mM	406.3 ± 55.8
			50 mM	180.6 ± 10.0
			25 mM	133.3 ± 3.9
4	**8b**	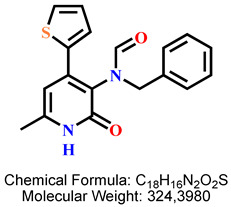	100 mM	1648.6 ± 179.5
			50 mM	136.0 ± 15.3
			25 mM	112.2 ± 6.8
5	**8c**	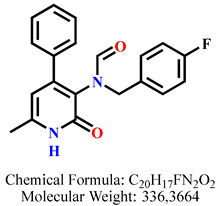	100 mM	179.9 ± 20.2
			50 mM	118.5 ± 4.7
			25 mM	116.2 ± 9.6
6	**8d**	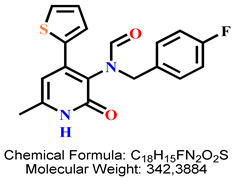	100 mM	2652.3 ± 134.3
			50 mM	191.9 ± 22.1
			25 mM	140.5 ± 10.3
7	**9a**	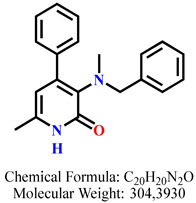	100 mM	112.6 ± 16.1
			50 mM	108.1 ± 3.4
			25 mM	108.6 ± 3.2
8	**9b**	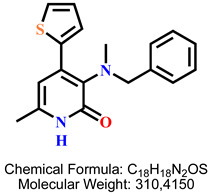	100 mM	498.2 ± 45.8
			50 mM	197.3 ± 18.2
			25 mM	128.4 ± 2.1
9	**9c**	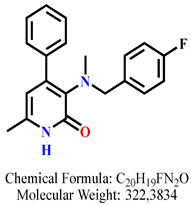	100 mM	573.9 ± 58.8
			50 mM	155.9 ± 16.1
			25 mM	99.5 ± 2.0
10	**9d**	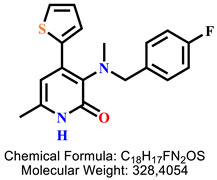	100 mM	209.0 ± 8.9
			50 mM	173.0 ± 9.0
			25 mM	99.5 ± 5.9
		Control		100.0

## Data Availability

The original contributions presented in the study are included in the article and Supplementary Material. Further inquiries can be directed to the corresponding authors.

## Supplementary Materials

The following supporting information can be downloaded at: https://www.mdpi.com/article/10.3390/molecules30163331/s1, Figure S1–S12: copies of NMR Spectra of compounds; Figure S13–S19: copies of Chromatograms and Mass Spectra of compounds; Figure S20: X-ray crystal structure of compounds **6**, **8a**, **8c**, **9a**.
