# Supplementary material for: Synthesis, Structure and Cytoprotective Activity of New Derivatives of 4-Aryl-3-Aminopyridin-2(1H)-One
Source: Molecules. 2025 Aug 9;30(16):3331. doi: 10.3390/molecules30163331 (PMC12388465; doi:10.3390/molecules30163331)
Supplement: Supplementary file 1 [file molecules-30-03331-s001.zip › molecules-3763451-supplementary.pdf]

Supporting Information

Synthesis, Structure and Cytoprotective Activity of New Derivatives of 4-Aryl-3-Aminopyridin-2(1*H*)-One

Zarina Shulgau [1,2,3,*], Irina Palamarchuk [4], Egor Dezhko [4], Shynggys Sergazy [1,2], Assel Urazbayeva [1], Yuliya Safarova [1], Alexander Gulyayev [1,2], Yuri Gatilov [5] and Ivan Kulakov [1,4,*]

1 National Laboratory Astana, Nazarbayev University, Kabanbai Batyr ave. 53, Z05H0P9 Astana, Kazakhstan; shynggys.sergazy@gmail.com (S.S.); assel.urazbayeva@nu.edu.kz (A.U.); yantsen@nu.edu.kz (Y.S.); akin@mail.ru (A.G.)
2 CF "Institute of Innovative and Preventive Medicine", Alikhan Bokeikhan street, building 1, Z05M0M4, Astana, Kazakhstan; zarina.shulgau@icloud.com (Z.S.); akin@mail.ru (A.G.); shynggys.sergazy@gmail.com (S.S.)
3 Department of Science Development, "Astana Medical University", Beibitshilik St 49/A, Z10K9D9, Astana, Kazakhstan; zarina.shulgau@icloud.com (Z.S.)
4 School of Natural Sciences, Tyumen State University, 15a Perekopskaya St., Tyumen 625003, Russia; i.v.palamarchuk@utmn.ru (I.P.)
5 N.N. Vorozhtsov Novosibirsk Institute of Organic Chemistry, Siberian Branch of the Russian Academy of Science, 9 Akademika Lavrientieva Ave., Novosibirsk 630090, Russia; gatilov@nioch.nsc.ru
* Correspondence: zarina.shulgau@icloud.com (Z.S.); i.v.kulakov@utmn.ru (I.K.); Tel.: +7 777 708 0837 (Z.S.).

Table of Contents

1. Materials and Methods 1
2. Copies of NMR Spectra of Products 2
3. Copies of Chromatograms and Mass Spectra of Products 14
4. X-Ray Structural Study of Product 17

1. Materials and Methods

The description of this section (figures of spectrums) is included as supplementary material.

^{1}H and ^{13}C NMR spectra were recorded on a Bruker DRX400 («Bruker BioSpin GmbH», Germany) (400 and 100 MHz, respectively), Bruker AVANCE 500 («Bruker BioSpin GmbH», Germany) (500 and 125 MHz, respectively), and Magritek spinsolve 80 carbon ultra (Aachen, Germany) (81 and 20 MHz, respectively) instruments using DMSO-d$_6$ or $CDCl_3$. The internal standard was residual solvent signals (7.25 and 77.0 ppm ^{1}H and for ^{13}C nuclei in $CDCl_3$ and 2.49 and 39.9 ppm 1H and for 13C nuclei in DMSO-d6). Elemental analysis was performed with a Carlo Erba 1106 CHN analyzer (Milan, Italy). Melting points were determined using a Stuart SMP10 (Stuart, United Kingdom) hot bench. Monitoring of the reaction course and the purity of the products was carried out by TLC (Merck, Darmstadt, Germany) on Sorbfil plates and visualized using iodine vapor or UV light.

Chromatographic mass spectrometric studies were performed on a Trace GC Ultra chromatograph with a DSQ II mass-selective detector in the electron ionization mode (70 eV) on a Thermo TR-5 MS quartz capillary column, 15 m long, 0.25 mm internal diameter, with a stationary phase film thickness of 0.25 µm. Splitless injection mode was used. The carrier gas discharge was 20 ml/min. The carrier gas (helium) velocity was 1 ml/min. The injector temperature was 200°C, transition chamber temperature 200°C was, and ion source temperature 200°C. Column thermostat temperature was changed according to the program from 40oC (delay from 2 to 5 min) to 220oC at a rate of 20oC per min, to 290°C at a rate of 15°C per min. The total analysis time was 30 min. The volume of the injected sample was 1 µl. Chromatograms were recorded in TIC mode. The mass scanning range was 30–500 amu.

Copies of NMR Spectra of Products

No.	(ppm)	(Hz)	Height	No.	(ppm)	(Hz)	Height	No.	(ppm)	(Hz)	Height	No.	(ppm)	(Hz)	Height
1	2.13	850.7	0.9011	9	6.87	2745.8	0.0996	17	7.15	2860.3	1.0000	25	7.42	2967.1	0.0767
2	4.16	1664.0	0.2174	10	6.92	2767.2	0.1787	18	7.17	2866.4	0.2207	26	7.86	3142.6	0.1625
3	4.23	1693.0	0.5396	11	6.94	2776.3	0.2064	19	7.19	2875.5	0.1931	27	7.88	3148.7	0.1621
4	5.77	2306.4	0.3528	12	7.01	2802.3	0.1750	20	7.21	2881.6	0.3463	28	10.42	4166.4	0.1799
5	6.80	2716.8	0.2588	13	7.03	2809.9	0.2046	21	7.23	2889.3	0.2552	29	12.91	5162.8	0.0365
6	6.81	2724.5	0.3214	14	7.07	2828.2	0.0637	22	7.27	2906.0	0.1881				
7	6.83	2730.6	0.0973	15	7.09	2835.8	0.1626	23	7.39	2953.3	0.0846				
8	6.85	2738.2	0.1737	16	7.11	2843.5	0.1303	24	7.40	2959.4	0.1258				

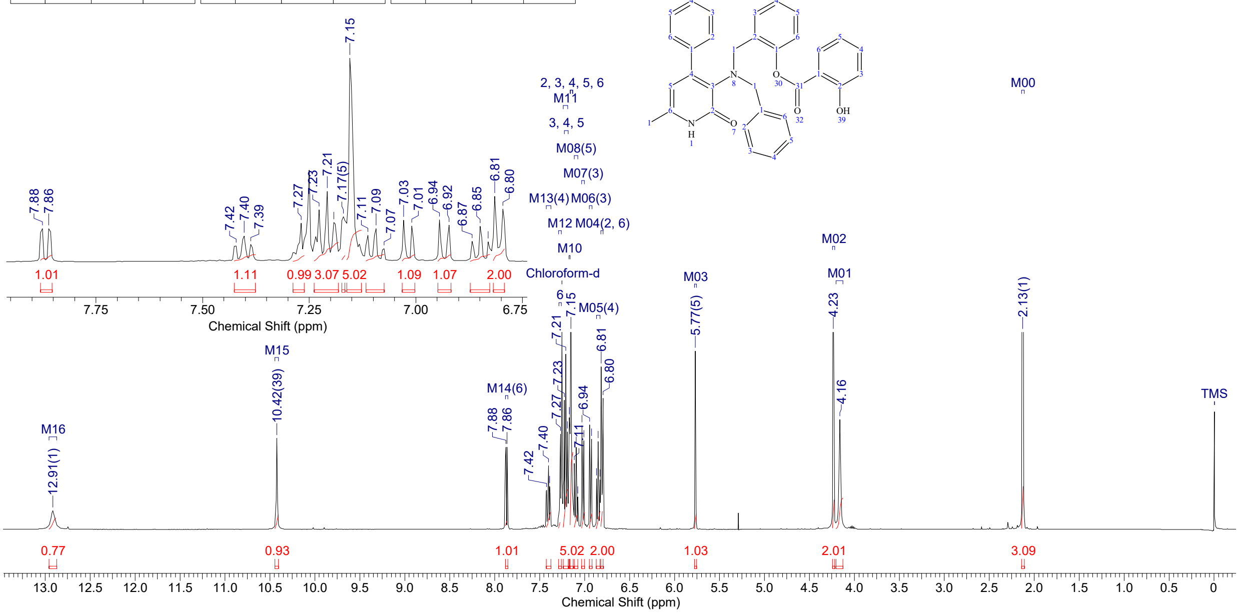

No.	(ppm)	(Hz)	Height	No.	(ppm)	(Hz)	Height	No.	(ppm)	(Hz)	Height	No.	(ppm)	(Hz)	Height
1	18.21	1830.5	0.0926	9	126.25	12690.9	0.0754	17	130.44	13112.7	0.0846	25	148.51	14928.9	0.0617
2	49.05	4931.2	0.0629	10	126.76	12742.7	0.0788	18	131.62	13231.5	0.0741	26	150.84	15162.8	0.0528
3	56.77	5706.6	0.0731	11	127.30	12797.3	0.0733	19	131.75	13244.0	0.0655	27	162.02	16287.1	0.0766
4	108.52	10909.1	0.0742	12	127.55	12822.2	0.1705	20	133.10	13380.1	0.0533	28	164.41	16527.7	0.0586
5	111.68	11226.4	0.0576	13	127.73	12840.4	0.0827	21	136.10	13682.0	0.0724	29	168.68	16957.1	0.0545
6	117.55	11816.8	0.0806	14	128.00	12867.3	0.1793	22	139.18	13991.6	0.0677				
7	119.23	11985.5	0.0852	15	128.35	12902.8	0.1669	23	139.24	13997.3	0.0572				
8	121.75	12238.5	0.0802	16	129.05	12972.7	0.1760	24	140.78	14151.6	0.0564				

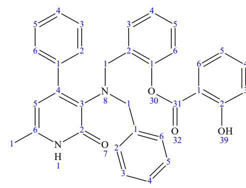

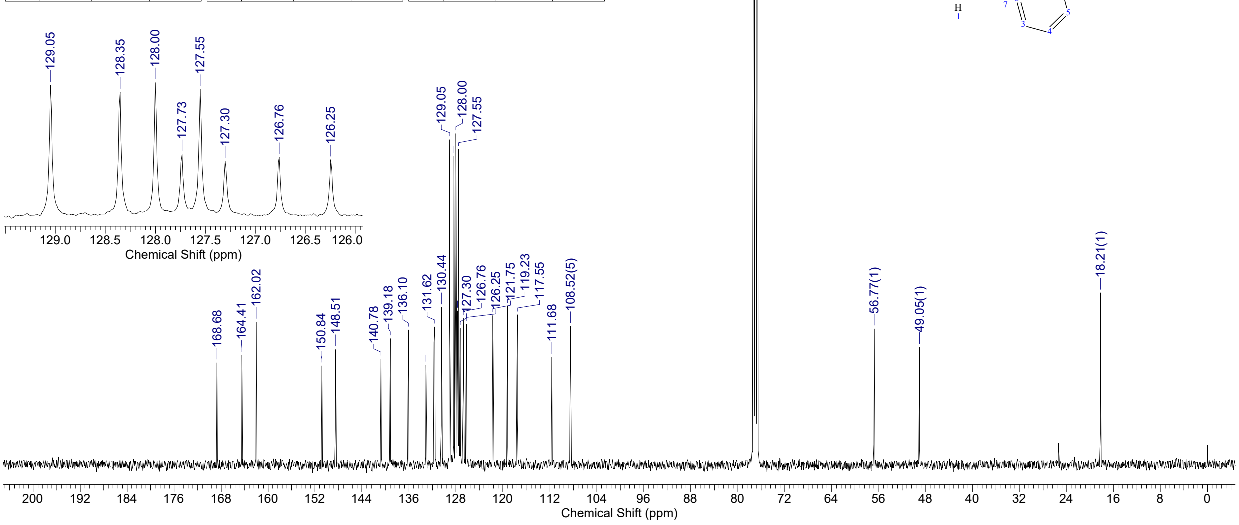

Figure S1. ^{1}H (400 MHz, $CDCl_3$) and ^{13}C (100 MHz, $CDCl_3$) NMR Spectra of **6**

No.	(ppm)	(Hz)	Height
1	2.20	176.8	0.7152
2	6.00	483.3	0.2347
3	7.38	593.9	1.0000
4	7.95	640.3	0.1528
5	8.64	695.4	0.0294
6	8.78	706.6	0.0248
7	9.11	733.3	0.0743
8	11.91	958.8	0.0957

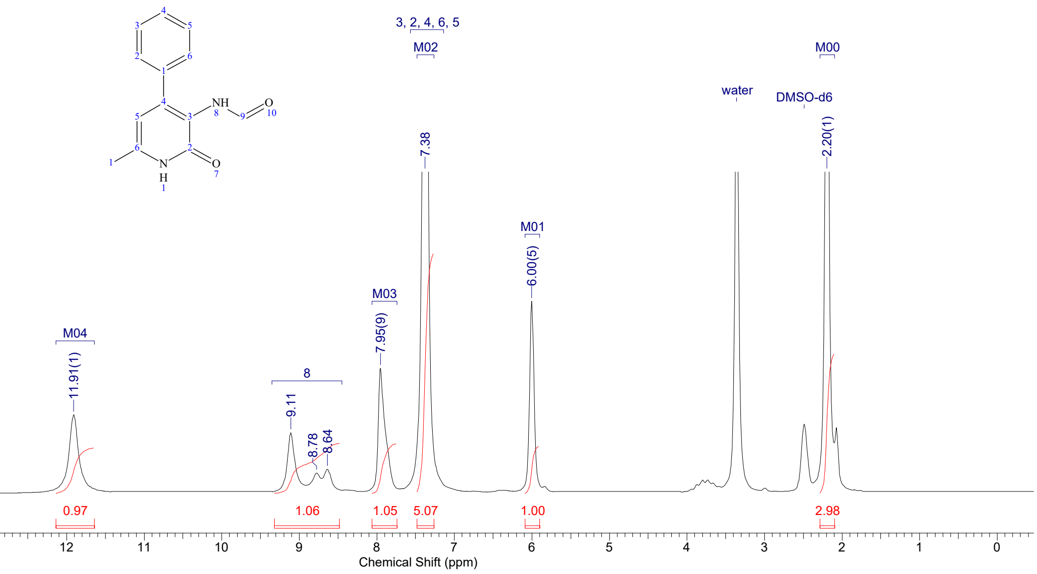

No.	(ppm)	(Hz)	Height
1	18.34	371.3	0.2020
2	105.96	2144.9	0.0506
3	119.97	2428.7	0.0294
4	127.60	2583.1	0.1078
5	128.25	2596.2	0.3182

No.	(ppm)	(Hz)	Height
6	136.99	2773.2	0.0325
7	137.76	2788.8	0.0310
8	143.04	2895.8	0.0491
9	160.50	3249.1	0.0810
10	164.86	3337.5	0.0451

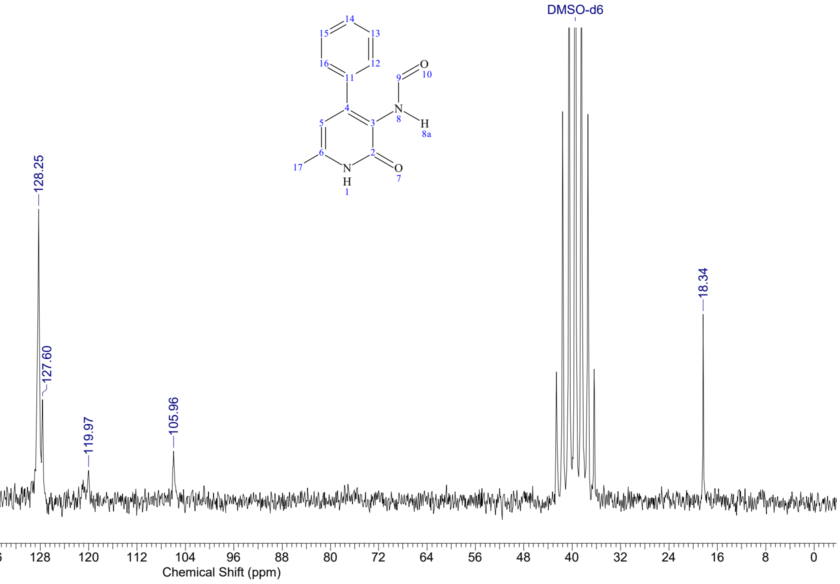

Figure S2. ^{1}H (81 MHz, DMSO-d6) and ^{13}C (20 MHz, DMSO-d6) NMR Spectra of **7a**

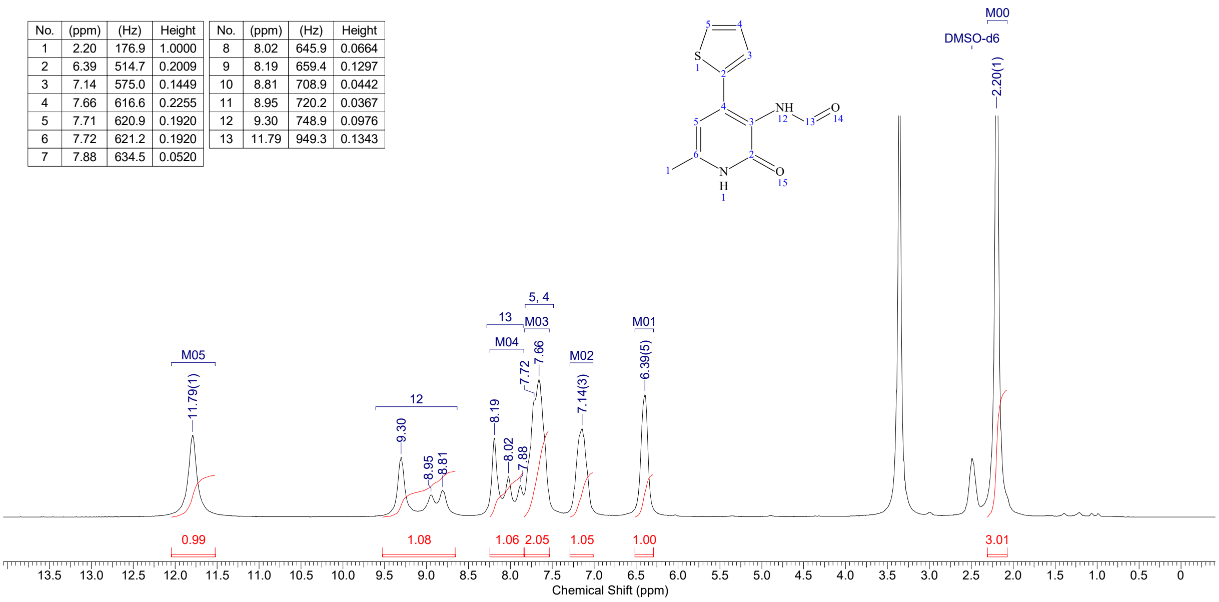

No.	(ppm)	(Hz)	Height
1	2.20	176.9	1.0000
2	6.39	514.7	0.2009
3	7.14	575.0	0.1449
4	7.66	616.6	0.2255
5	7.71	620.9	0.1920
6	7.72	621.2	0.1920
7	7.88	634.5	0.0520
8	8.02	645.9	0.0664
9	8.19	659.4	0.1297
10	8.81	708.9	0.0442
11	8.95	720.2	0.0367
12	9.30	748.9	0.0976
13	11.79	949.3	0.1343

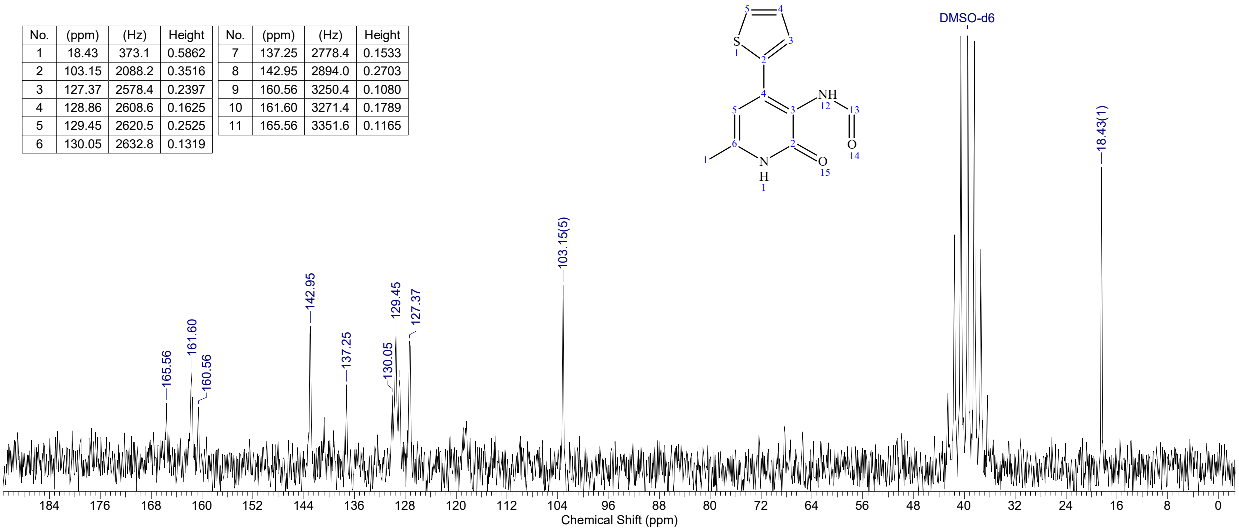

No.	(ppm)	(Hz)	Height
1	18.43	373.1	0.5862
2	103.15	2088.2	0.3516
3	127.37	2578.4	0.2397
4	128.86	2608.6	0.1625
5	129.45	2620.5	0.2525
6	130.05	2632.8	0.1319
7	137.25	2778.4	0.1533
8	142.95	2894.0	0.2703
9	160.56	3250.4	0.1080
10	161.60	3271.4	0.1789
11	165.56	3351.6	0.1165

Figure S3. ^{1}H (81 MHz, DMSO-d6) and ^{13}C (20 MHz, DMSO-d6) NMR Spectra of **7b**

No.	(ppm)	(Hz)	Height
1	2.17	174.6	0.9372
2	4.34	349.2	0.1522
3	5.95	478.6	0.2660
4	6.86	552.4	0.1087
5	6.88	554.1	0.1247
6	6.99	562.4	0.3554
7	7.03	566.2	0.4825
8	7.05	567.3	0.4861
9	7.09	570.6	0.3467

No.	(ppm)	(Hz)	Height
10	7.13	573.9	0.4614
11	7.17	577.0	0.3413
12	7.21	580.7	0.2632
13	7.29	587.0	0.3568
14	7.34	591.1	0.3269
15	7.37	593.4	0.2614
16	8.10	652.4	0.3623
17	12.02	967.6	0.1108

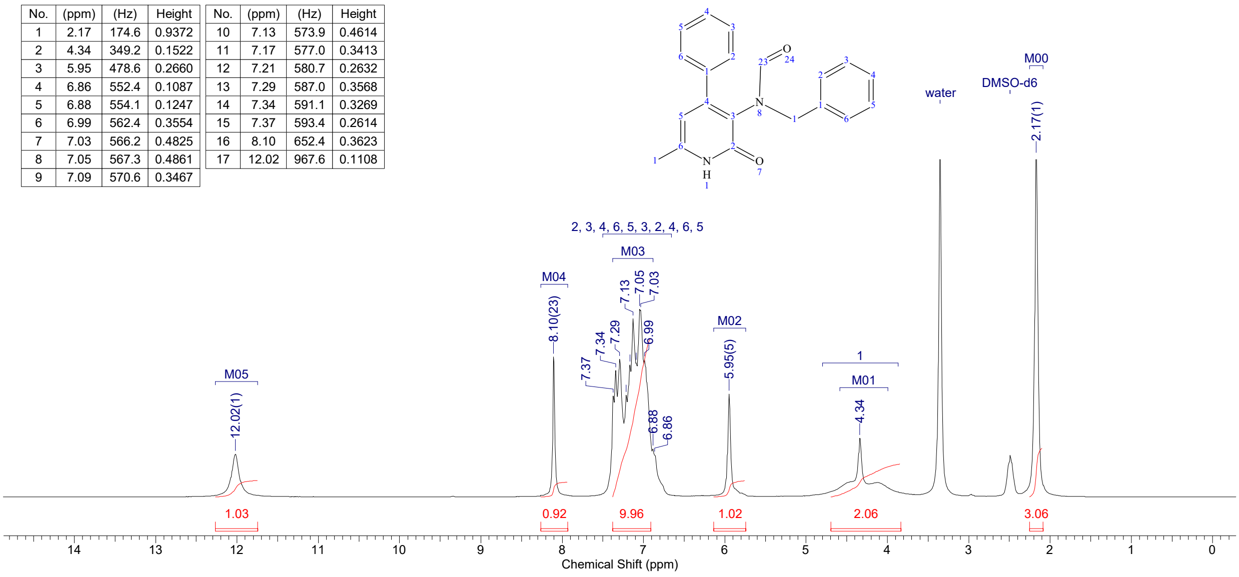

No.	(ppm)	(Hz)	Height
1	18.33	371.1	0.1794
2	46.31	937.6	0.1143
3	106.30	2151.9	0.1161
4	123.57	2501.5	0.1050
5	127.11	2573.2	0.1732
6	127.50	2581.1	0.3178
7	128.02	2591.5	0.4144

No.	(ppm)	(Hz)	Height
8	128.53	2601.9	0.5616
9	136.29	2759.1	0.1482
10	136.75	2768.3	0.1497
11	144.46	2924.4	0.1570
12	149.63	3029.0	0.1283
13	161.06	3260.4	0.1309
14	164.37	3327.4	0.1344

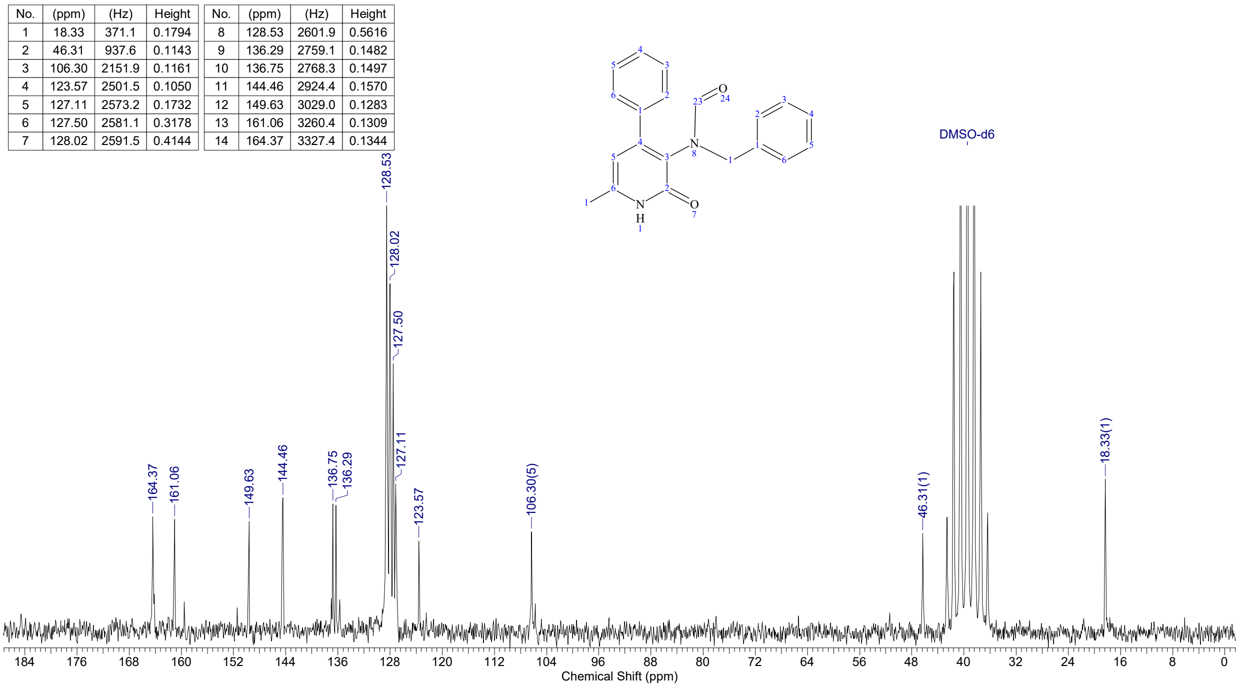

Figure S4. ^{1}H (81 MHz, DMSO-d6) and ^{13}C (20 MHz, DMSO-d6) NMR Spectra of **8a**

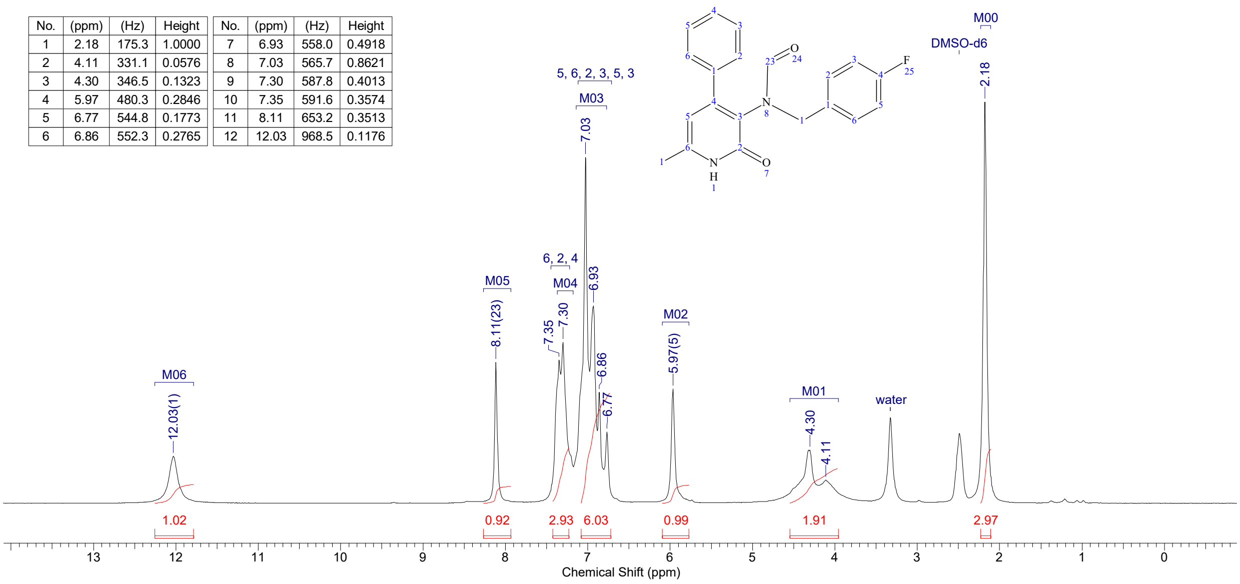

No.	(ppm)	(Hz)	Height	No.	(ppm)	(Hz)	Height
1	2.18	175.3	1.0000	7	6.93	558.0	0.4918
2	4.11	331.1	0.0576	8	7.03	565.7	0.8621
3	4.30	346.5	0.1323	9	7.30	587.8	0.4013
4	5.97	480.3	0.2846	10	7.35	591.6	0.3574
5	6.77	544.8	0.1773	11	8.11	653.2	0.3513
6	6.86	552.3	0.2765	12	12.03	968.5	0.1176

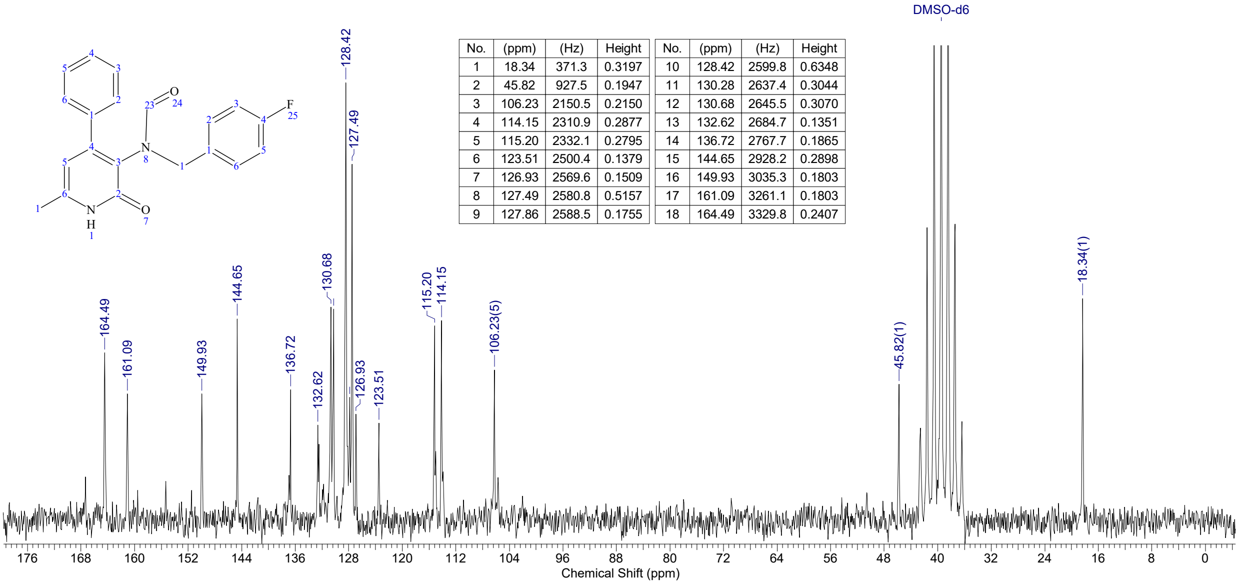

No.	(ppm)	(Hz)	Height	No.	(ppm)	(Hz)	Height
1	18.34	371.3	0.3197	10	128.42	2599.8	0.6348
2	45.82	927.5	0.1947	11	130.28	2637.4	0.3044
3	106.23	2150.5	0.2150	12	130.68	2645.5	0.3070
4	114.15	2310.9	0.2877	13	132.62	2684.7	0.1351
5	115.20	2332.1	0.2795	14	136.72	2767.7	0.1865
6	123.51	2500.4	0.1379	15	144.65	2928.2	0.2898
7	126.93	2569.6	0.1509	16	149.93	3035.3	0.1803
8	127.49	2580.8	0.5157	17	161.09	3261.1	0.1803
9	127.86	2588.5	0.1755	18	164.49	3329.8	0.2407

Figure S5. ^{1}H (81 MHz, DMSO-d6) and ^{13}C (20 MHz, DMSO-d6) NMR Spectra of **8c**

No.	(ppm)	(Hz)	Height
1	2.17	174.4	0.5596
2	4.09	329.3	0.0235
3	4.27	343.4	0.0583
4	4.70	378.7	0.0575
5	4.88	392.9	0.0235
6	6.29	506.7	0.1571
7	7.11	572.3	1.0000
8	7.33	590.5	0.1054
9	7.37	593.2	0.0800
10	7.70	620.2	0.0969
11	7.77	625.2	0.0825
12	8.07	649.7	0.2112
13	11.89	956.9	0.0735

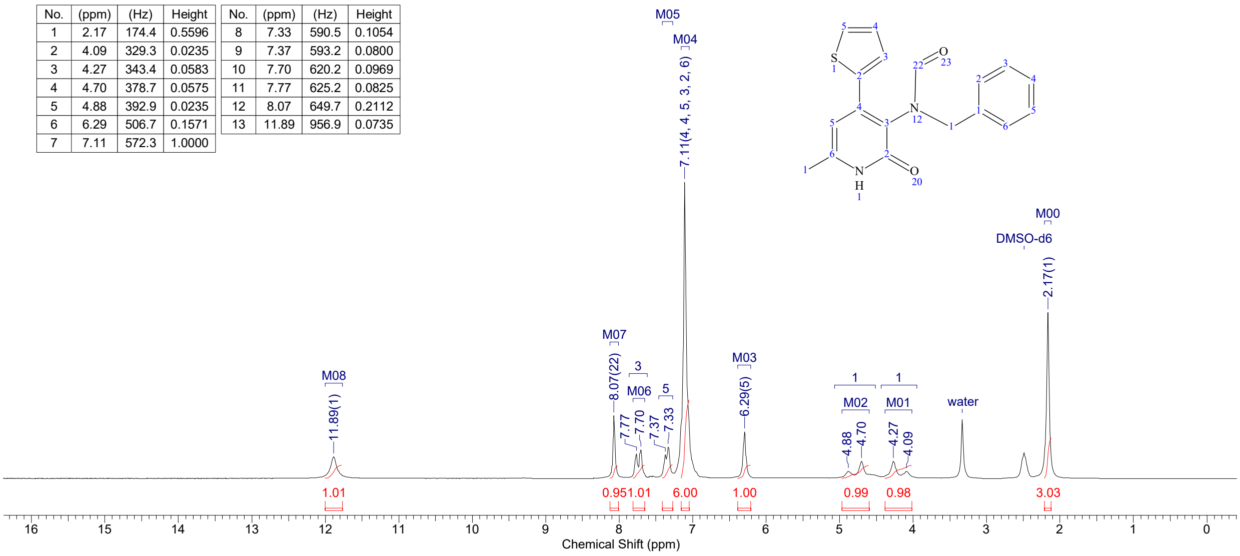

No.	(ppm)	(Hz)	Height
1	18.40	372.5	0.3633
2	47.36	958.8	0.2679
3	104.12	2107.8	0.2823
4	121.95	2468.8	0.1456
5	127.16	2574.2	0.3824
6	127.45	2580.0	0.3812
7	127.75	2586.2	0.9190
8	129.03	2612.0	0.9328
9	129.27	2617.0	0.3761
10	130.05	2632.7	0.3181
11	135.80	2749.2	0.2255
12	136.36	2760.4	0.1870
13	142.17	2878.0	0.1979
14	144.58	2926.8	0.2882
15	161.30	3265.4	0.2466
16	164.62	3332.6	0.2914

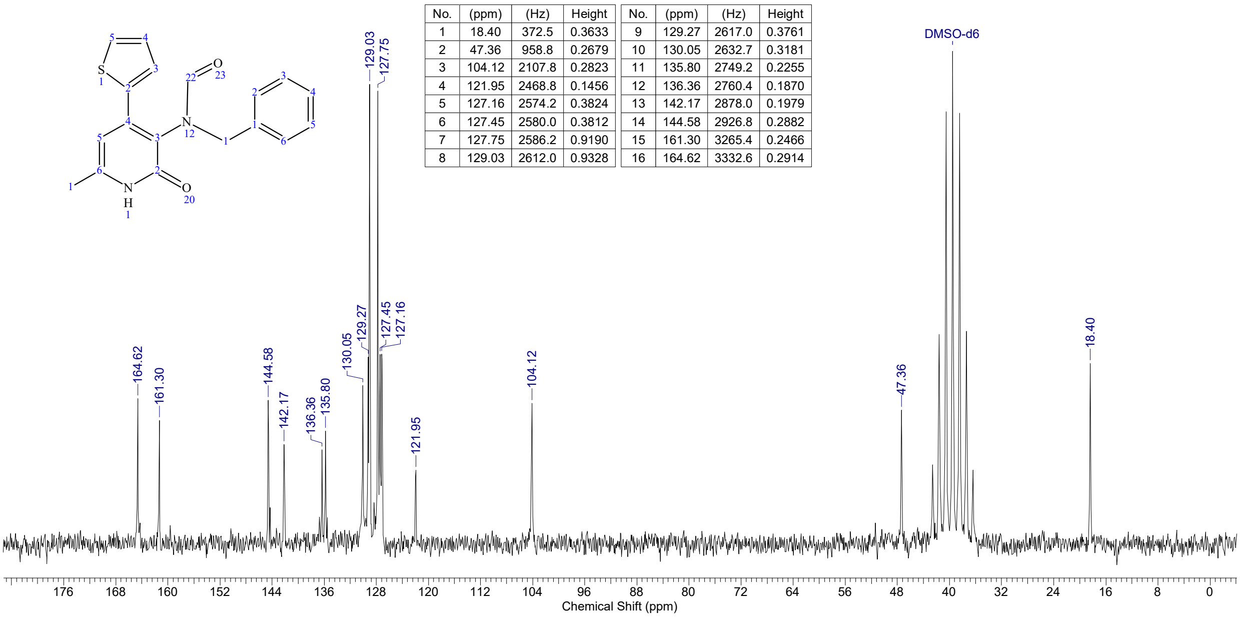

Figure S6. ^{1}H (81 MHz, DMSO-d6) and ^{13}C (20 MHz, DMSO-d6) NMR Spectra of **8b**

No.	(ppm)	(Hz)	Height	No.	(ppm)	(Hz)	Height
1	2.18	175.1	1.0000	9	6.93	558.0	0.3491
2	4.08	328.8	0.0666	10	7.04	566.7	0.5869
3	4.26	342.9	0.1486	11	7.13	573.8	0.5214
4	4.54	365.2	0.1252	12	7.39	594.8	0.2381
5	4.67	375.6	0.1488	13	7.72	621.7	0.1950
6	4.84	389.8	0.0666	14	8.06	648.7	0.3052
7	6.32	509.0	0.2898	15	11.90	958.2	0.1863
8	6.83	549.6	0.1453				

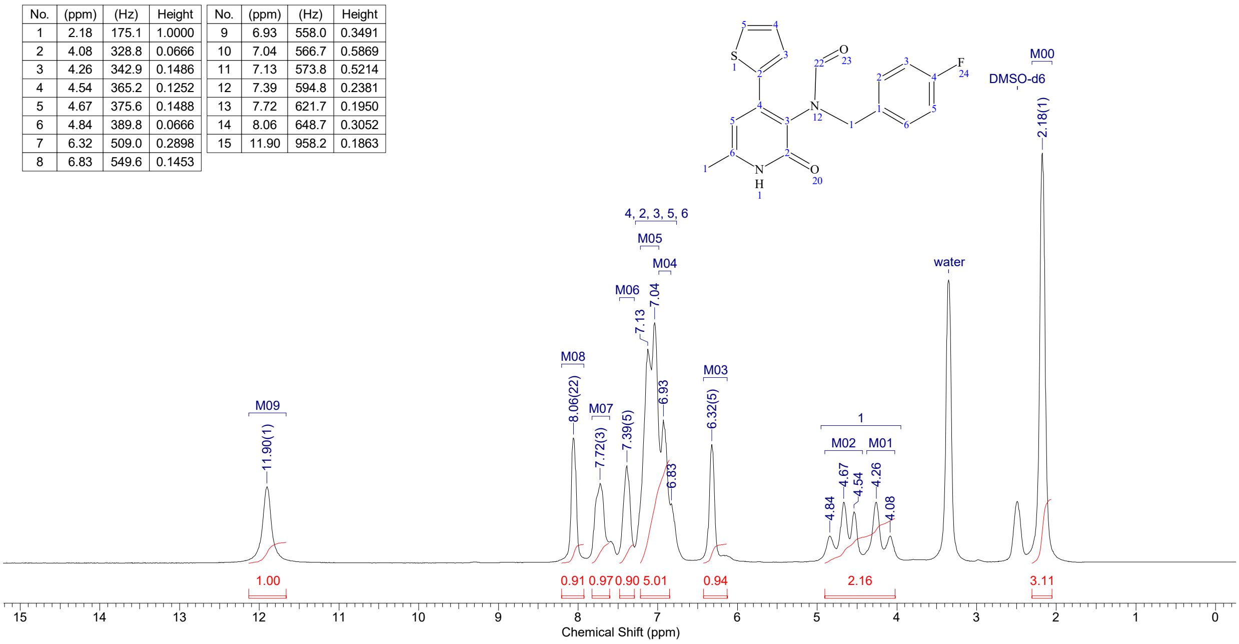

No.	(ppm)	(Hz)	Height	No.	(ppm)	(Hz)	Height
1	18.44	373.3	0.1869	11	131.32	2658.4	0.2219
2	46.75	946.3	0.1112	12	132.04	2673.0	0.0934
3	104.07	2106.8	0.1348	13	132.19	2675.9	0.0787
4	113.95	2306.7	0.2022	14	136.25	2758.2	0.1393
5	115.00	2328.0	0.1896	15	142.40	2882.8	0.1481
6	121.75	2464.8	0.1155	16	144.77	2930.8	0.1509
7	127.47	2580.5	0.1691	17	155.40	3145.8	0.1378
8	129.41	2619.7	0.1672	18	161.36	3266.5	0.1426
9	130.17	2635.1	0.1317	19	164.72	3334.5	0.1462
10	130.91	2650.1	0.2242				

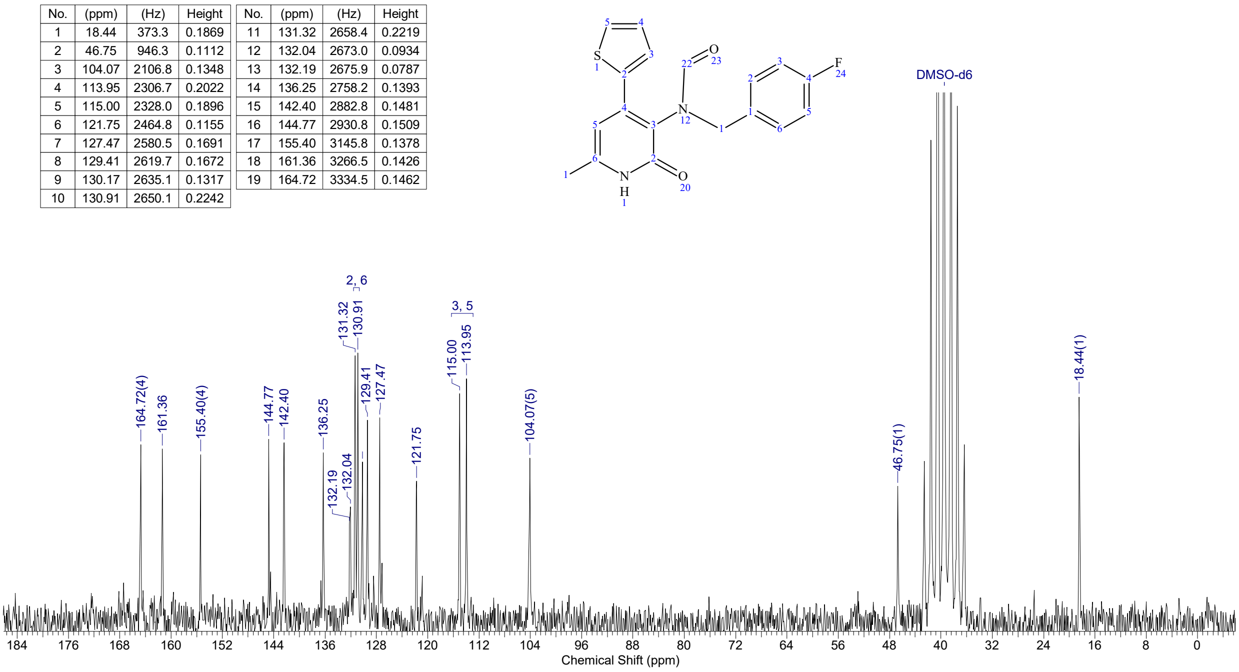

Figure S7. ^{1}H (81 MHz, DMSO-d6) and ^{13}C (20 MHz, DMSO-d6) NMR Spectra of **8d**

No.	(ppm)	(Hz)	Height	No.	(ppm)	(Hz)	Height	No.	(ppm)	(Hz)	Height
1	2.37	1183.0	0.7858	9	7.15	3573.9	0.0852	17	7.25	3625.9	0.1831
2	2.60	1300.9	1.0000	10	7.15	3576.5	0.3373	18	7.25	3628.1	0.1978
3	4.13	2065.0	0.5230	11	7.15	3578.3	0.3233	19	7.35	3676.8	0.0676
4	5.97	2983.8	0.2536	12	7.17	3583.6	0.1682	20	7.36	3682.2	0.3730
5	7.06	3529.6	0.1121	13	7.17	3587.1	0.0452	21	7.38	3689.5	0.1861
6	7.06	3531.1	0.1391	14	7.24	3618.9	0.1420	22	7.39	3695.1	0.0494
7	7.07	3536.0	0.1550	15	7.24	3620.3	0.1733	23	7.39	3698.3	0.0334
8	7.08	3538.6	0.1767	16	7.24	3622.8	0.1358	24	13.05	6524.5	0.0400

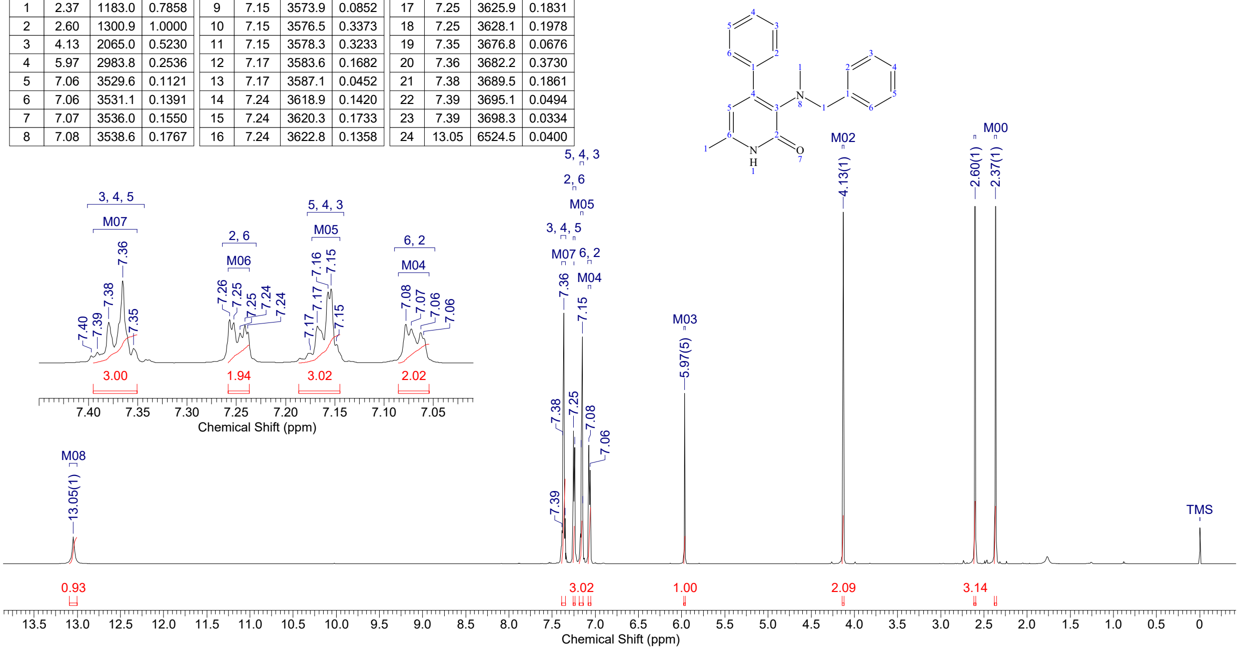

No.	(ppm)	(Hz)	Height	No.	(ppm)	(Hz)	Height
1	18.52	2329.6	0.4012	9	128.57	16169.3	0.9036
2	40.05	5037.0	0.4642	10	128.71	16186.3	0.9733
3	59.00	7419.1	0.4532	11	135.73	17068.7	0.1781
4	108.51	13646.1	0.3775	12	139.40	17531.1	0.2419
5	126.46	15903.4	0.4518	13	139.70	17568.1	0.2676
6	127.53	16037.5	0.3570	14	140.09	17617.4	0.2283
7	127.80	16072.2	0.9440	15	149.09	18749.5	0.2209
8	127.83	16076.1	1.0000	16	164.83	20728.6	0.2298

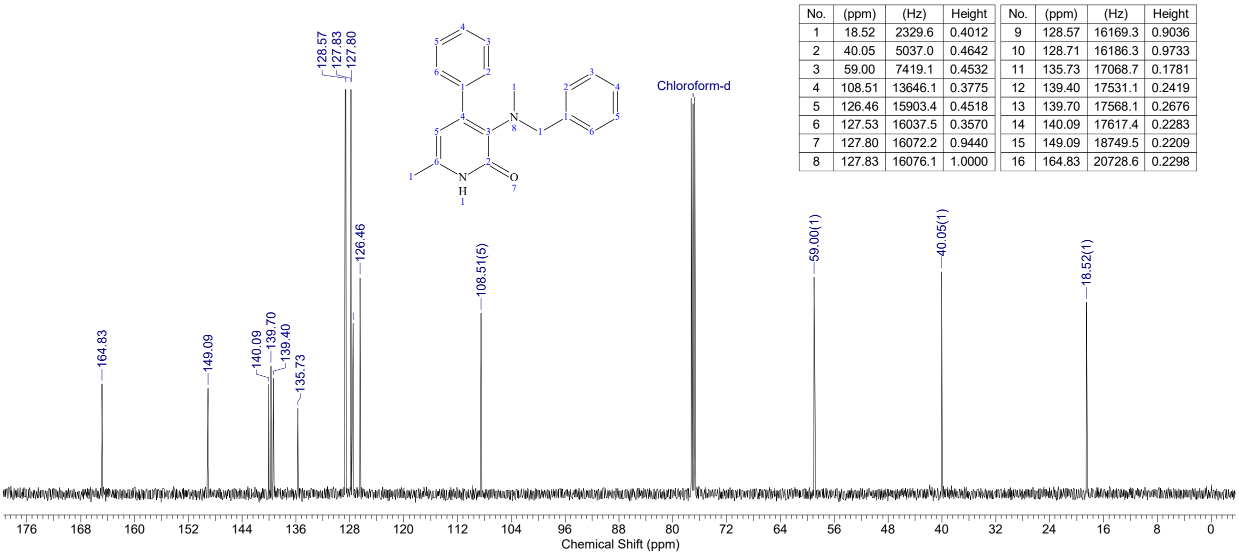

Figure S8. ^{1}H (500 MHz, $CDCl_3$) and ^{13}C (125 MHz, $CDCl_3$) NMR Spectra of **9a**

No.	(ppm)	(Hz)	Height	No.	(ppm)	(Hz)	Height	No.	(ppm)	(Hz)	Height
1	2.36	1180.9	0.7710	8	6.97	3487.6	0.1285	15	7.36	3682.5	0.0509
2	2.61	1306.6	1.0000	9	6.98	3493.2	0.1463	16	7.37	3683.5	0.0599
3	4.06	2031.9	0.4317	10	6.99	3495.9	0.1253	17	7.38	3689.1	0.3580
4	5.97	2985.8	0.2349	11	7.00	3501.8	0.1041	18	7.38	3691.1	0.1462
5	6.80	3402.3	0.1192	12	7.24	3619.7	0.1381	19	7.39	3696.2	0.1670
6	6.82	3411.1	0.2591	13	7.24	3621.1	0.1633	20	13.01	6506.7	0.0230
7	6.84	3419.9	0.1459	14	7.26	3628.5	0.2242				

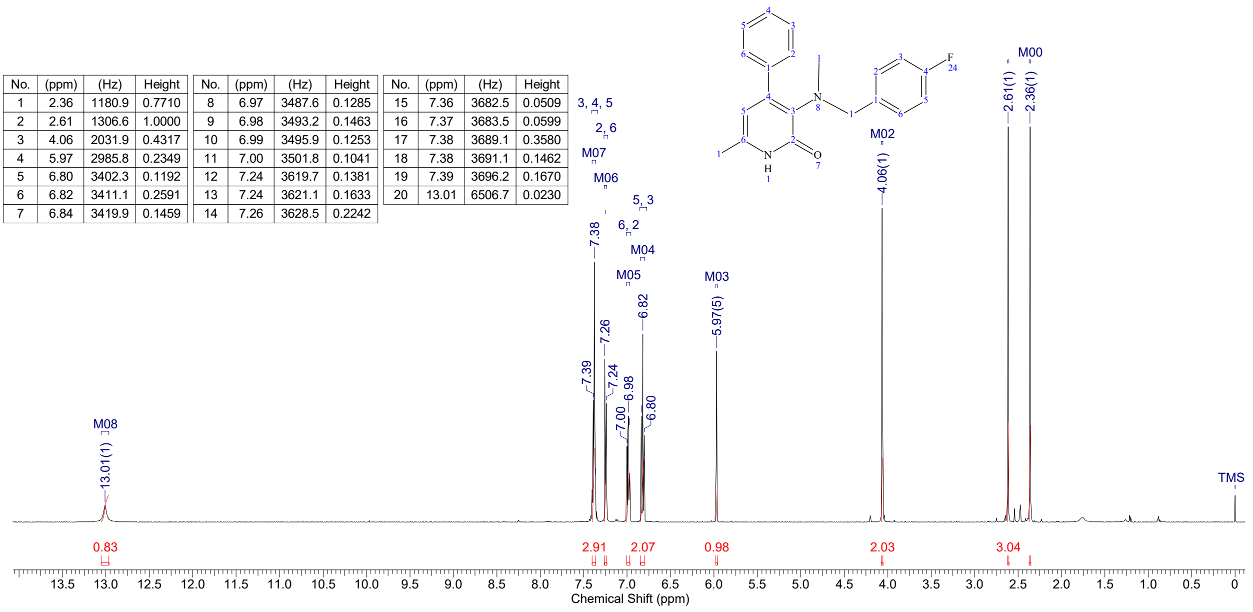

No.	(ppm)	(Hz)	Height	No.	(ppm)	(Hz)	Height	No.	(ppm)	(Hz)	Height
1	18.54	2331.2	0.5042	8	127.83	16075.3	1.0000	15	139.30	17518.0	0.2343
2	40.07	5038.5	0.5512	9	128.55	16166.2	0.9864	16	140.31	17645.1	0.2878
3	58.27	7327.4	0.4719	10	130.12	16363.5	0.5384	17	149.52	18803.5	0.2535
4	108.46	13640.0	0.4656	11	130.18	16371.2	0.5501	18	160.74	20213.8	0.1042
5	114.41	14387.5	0.4671	12	135.33	17018.6	0.1450	19	162.68	20458.1	0.1108
6	114.57	14408.3	0.5233	13	135.35	17021.7	0.1516	20	164.79	20724.0	0.2514
7	127.61	16048.3	0.4235	14	135.43	17031.7	0.2248				

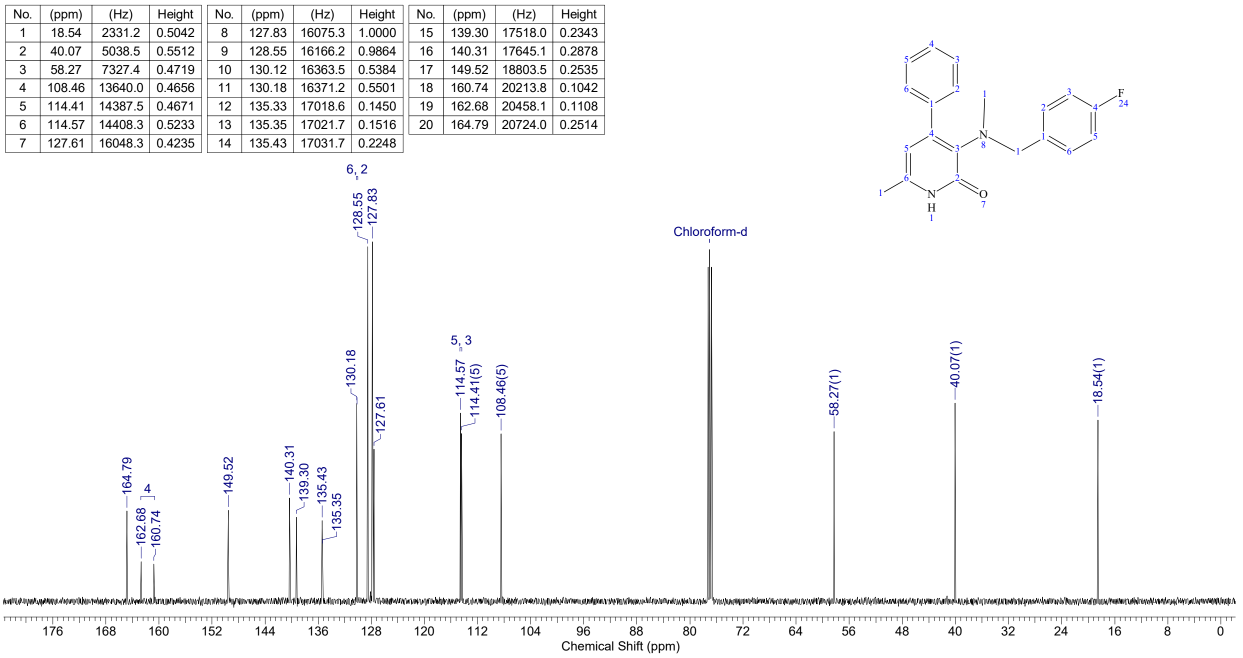

Figure S9. ^{1}H (400 MHz, $CDCl_3$) and ^{13}C (100 MHz, $CDCl_3$) NMR Spectra of **9c**

No.	(ppm)	(Hz)	Height
1	2.09	168.6	0.8445
2	2.60	209.1	0.8621
3	4.25	342.2	0.5387
4	6.37	513.0	0.2804
5	7.15	575.2	0.6465
6	7.60	612.0	0.3797
7	7.64	614.9	0.3057
8	11.36	914.7	0.1125

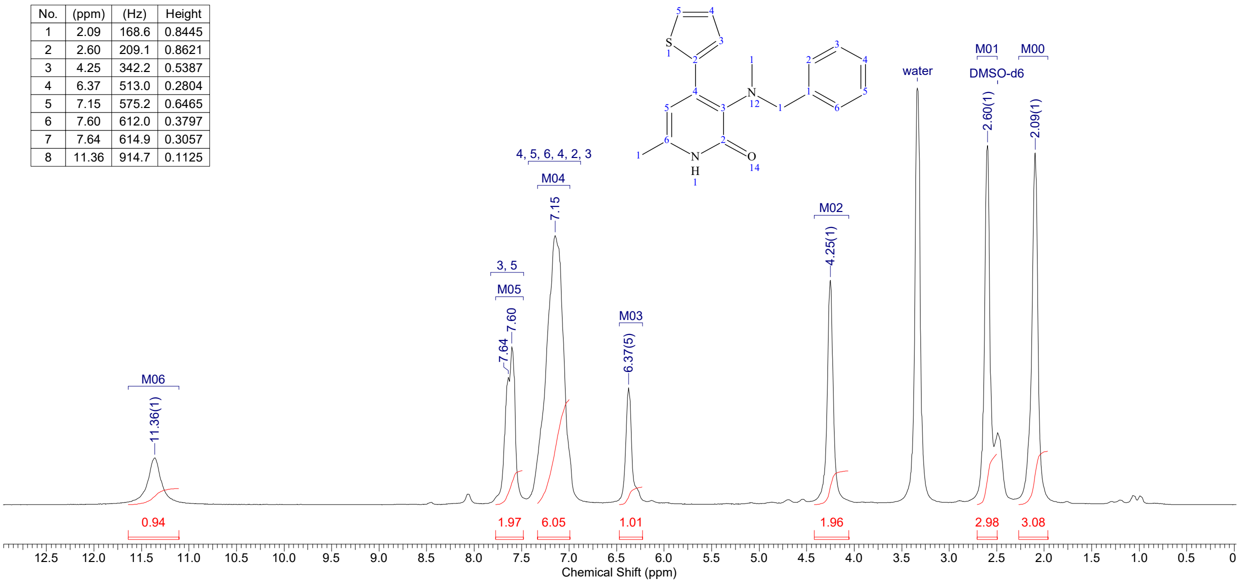

No.	(ppm)	(Hz)	Height	No.	(ppm)	(Hz)	Height
1	18.27	370.0	0.2561	9	129.70	2625.6	0.5692
2	39.91	807.9	0.3530	10	131.16	2655.2	0.1475
3	57.49	1163.9	0.2059	11	131.56	2663.3	0.2572
4	101.62	2057.3	0.2280	12	136.62	2765.8	0.1537
5	125.97	2550.2	0.2514	13	138.19	2797.5	0.2310
6	126.79	2566.7	0.2649	14	140.38	2841.8	0.1644
7	127.57	2582.6	0.6152	15	141.19	2858.3	0.2624
8	128.00	2591.3	0.2764	16	162.11	3281.7	0.1999

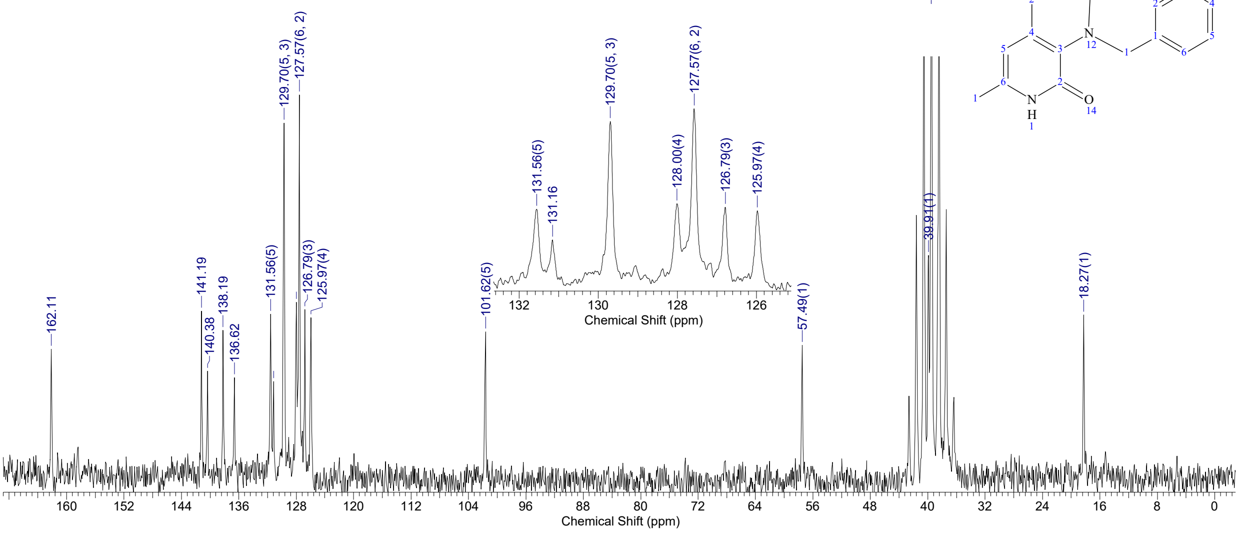

Figure S10. ^{1}H (81 MHz, DMSO-d6) and ^{13}C (20 MHz, DMSO-d6) NMR Spectra of **9b**

No.	(ppm)	(Hz)	Height	No.	(ppm)	(Hz)	Height
1	2.10	169.2	0.5929	8	7.11	572.2	0.1179
2	2.60	209.4	0.6285	9	7.19	578.4	0.1471
3	4.24	341.0	0.3558	10	7.26	584.4	0.1763
4	6.38	513.6	0.1961	11	7.36	592.2	0.0817
5	6.82	549.3	0.0819	12	7.60	611.4	0.2710
6	6.93	557.8	0.2021	13	7.65	615.6	0.2064
7	7.04	567.1	0.2399	14	11.36	914.7	0.0724

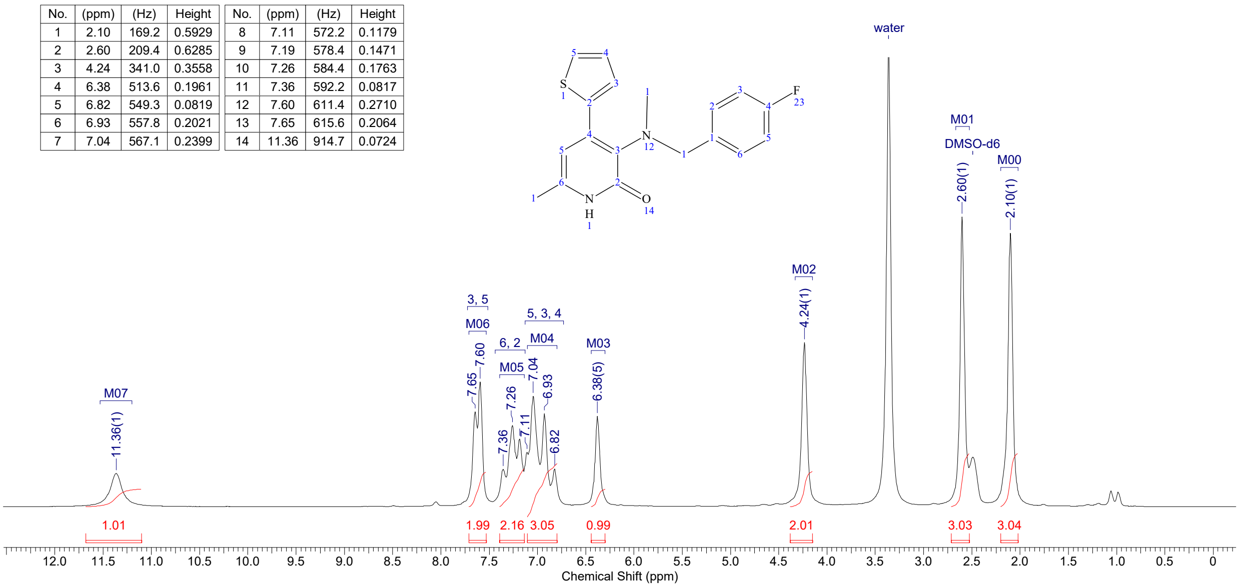

No.	(ppm)	(Hz)	Height	No.	(ppm)	(Hz)	Height
1	18.30	370.6	0.3744	11	131.74	2666.9	0.4842
2	39.96	808.9	0.4155	12	134.33	2719.4	0.1923
3	56.60	1145.8	0.2291	13	134.48	2722.3	0.1888
4	101.66	2058.0	0.2959	14	136.56	2764.5	0.2433
5	113.77	2303.1	0.3678	15	140.55	2845.3	0.2263
6	114.80	2324.1	0.3734	16	141.33	2861.1	0.3719
7	126.04	2551.6	0.3281	17	155.17	3141.3	0.0684
8	128.10	2593.3	0.3295	18	162.16	3282.8	0.3136
9	130.84	2648.7	0.1885	19	167.16	3384.0	0.0742
10	131.34	2658.9	0.4006				

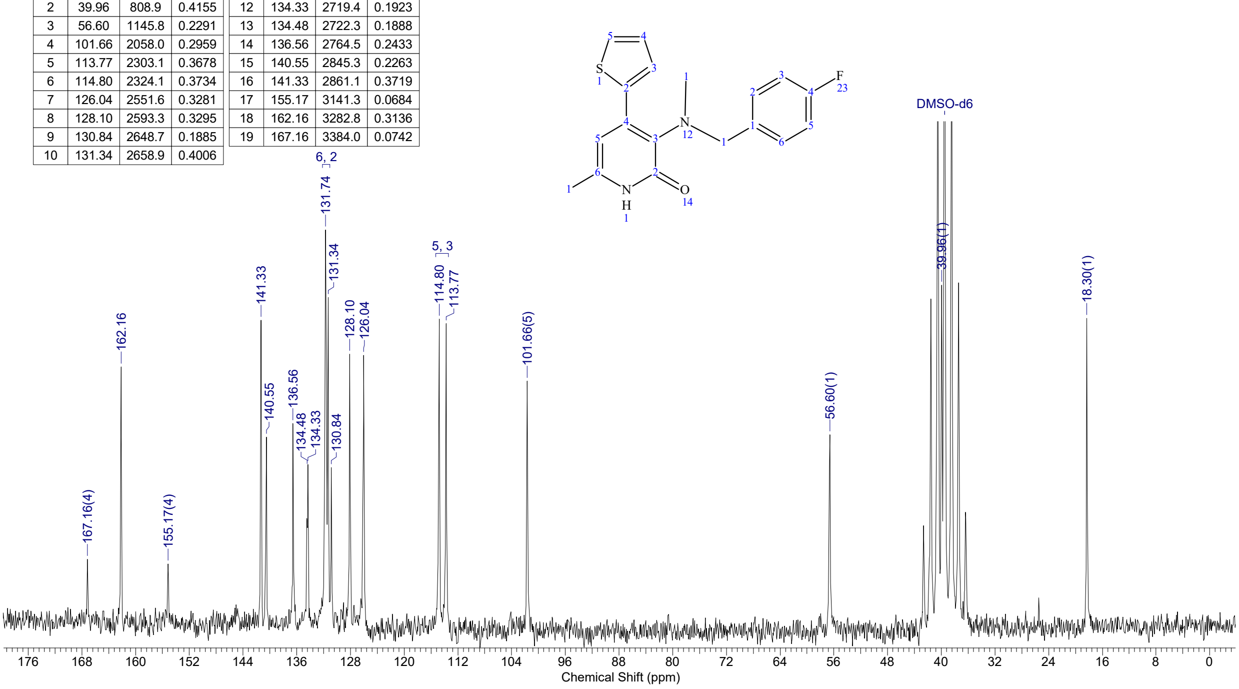

Figure S11. ^{1}H (81 MHz, DMSO-d6) and ^{13}C (20 MHz, DMSO-d6) NMR Spectra of **9d**

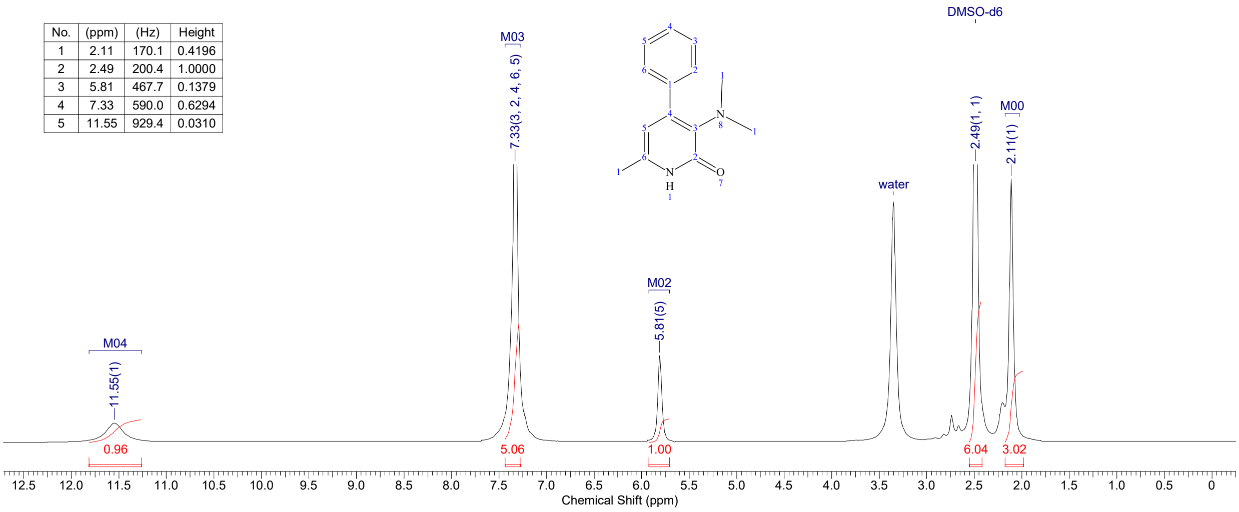

No.	(ppm)	(Hz)	Height
1	2.11	170.1	0.4196
2	2.49	200.4	1.0000
3	5.81	467.7	0.1379
4	7.33	590.0	0.6294
5	11.55	929.4	0.0310

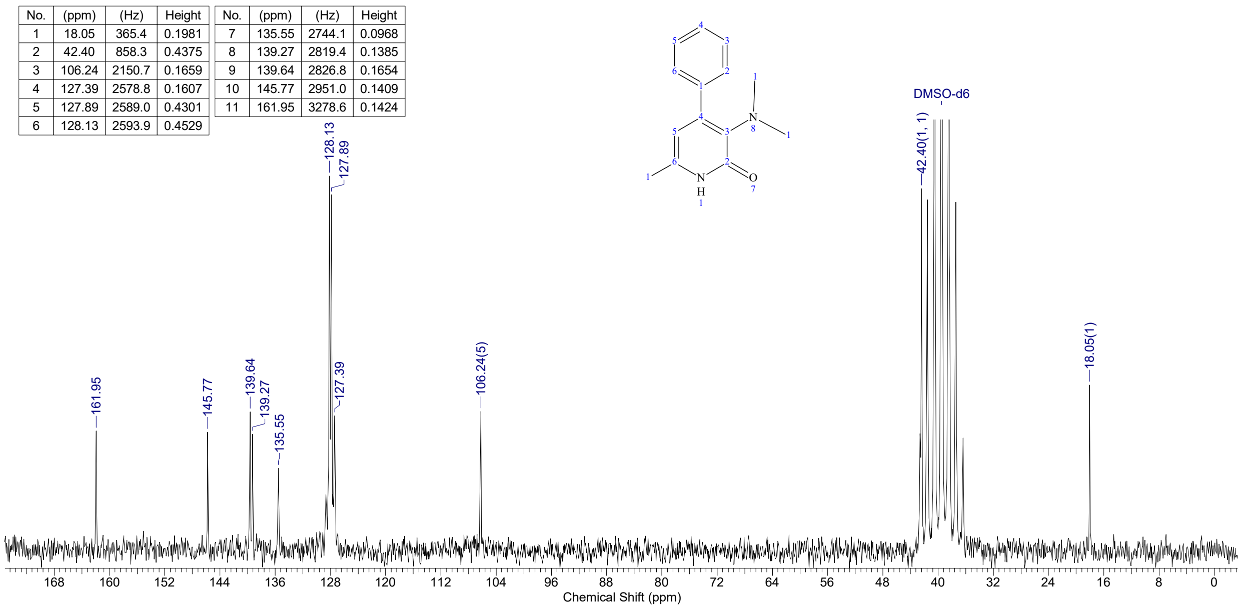

No.	(ppm)	(Hz)	Height
1	18.05	365.4	0.1981
2	42.40	858.3	0.4375
3	106.24	2150.7	0.1659
4	127.39	2578.8	0.1607
5	127.89	2589.0	0.4301
6	128.13	2593.9	0.4529

No.	(ppm)	(Hz)	Height
7	135.55	2744.1	0.0968
8	139.27	2819.4	0.1385
9	139.64	2826.8	0.1654
10	145.77	2951.0	0.1409
11	161.95	3278.6	0.1424

Figure S12. ^{1}H (81 MHz, DMSO-d6) and ^{13}C (20 MHz, DMSO-d6) NMR Spectra of **10**

Copies of Chromatograms and Mass Spectra of Products

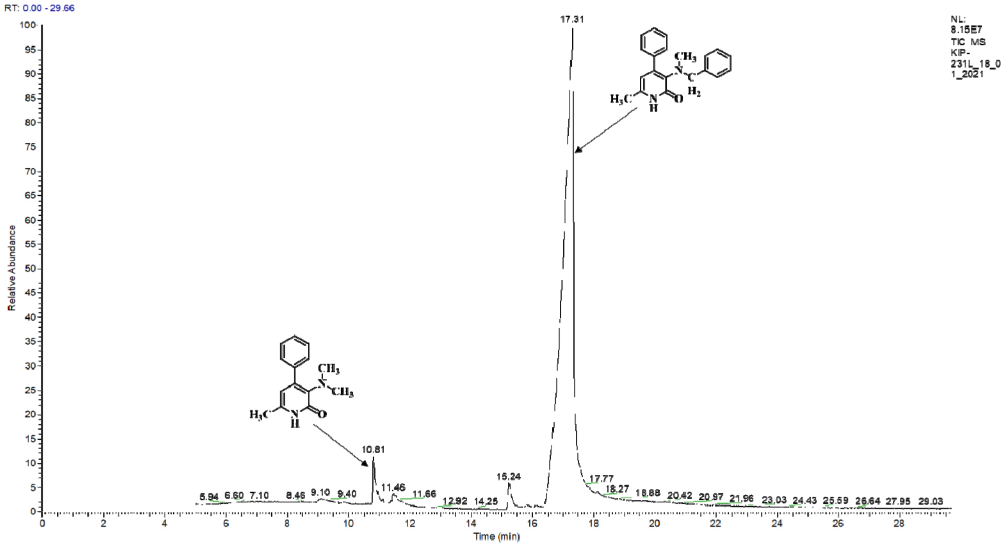

Figure S13. Chromatogram of the reaction mixture in the reaction of aminopyridone **2a** with formaldehyde and formic acid

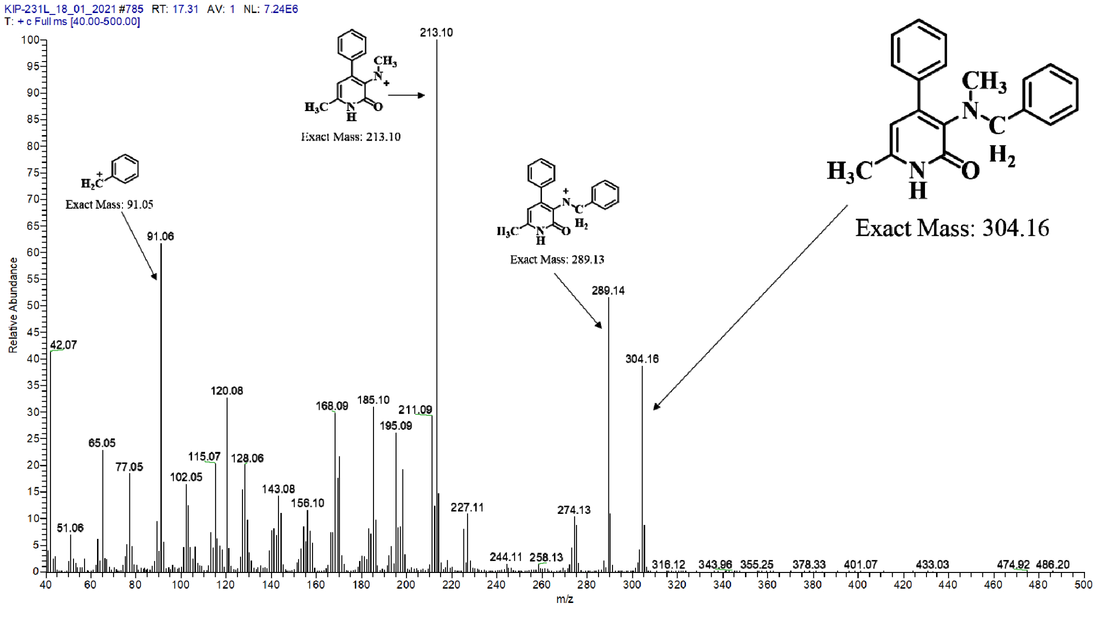

Figure S14. Mass spectrum of 3-(benzyl(methyl)amino)-6-methyl-4-phenylpyridin-2(1*H*)-one **9a**

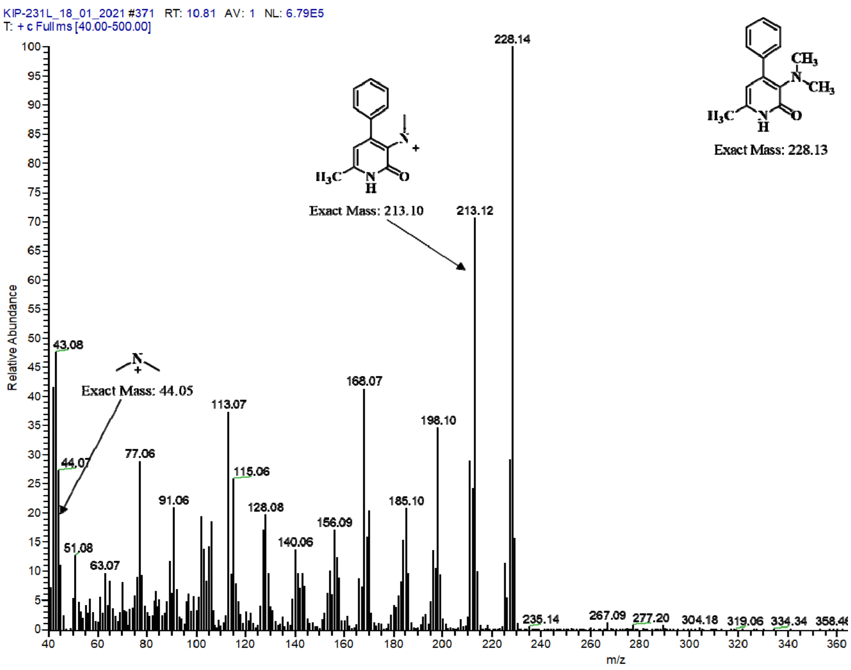

Figure S15. Mass spectrum of 3-(dimethylamino)-6-methyl-4-phenylpyridin-2(1*H*)-one **10**

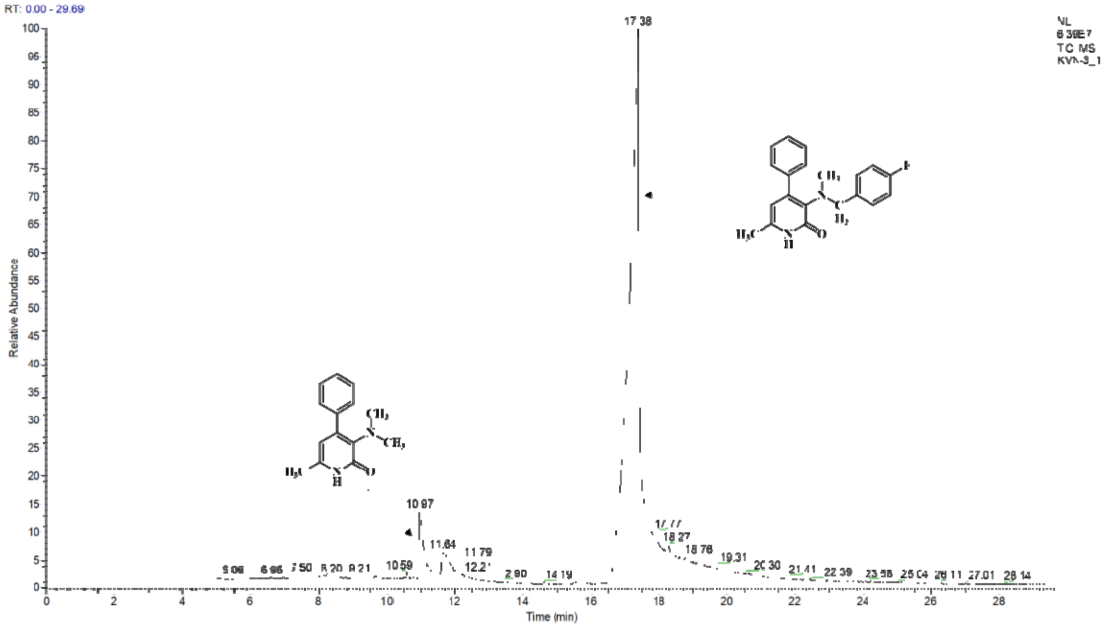

Figure S16. Chromatogram of the reaction mixture in the reaction of aminopyridone 2b with formaldehyde and formic acid

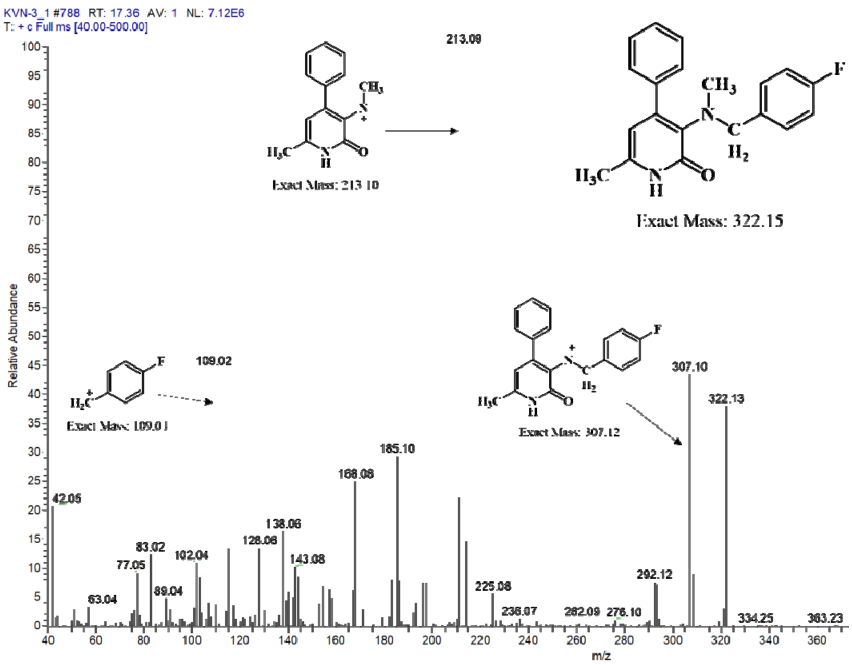

Figure S17. Mass spectrum of 3-((4-fluorobenzyl)(methyl)amino)-6-methyl-4-phenylpyridin-2(1*H*)-one **9b**

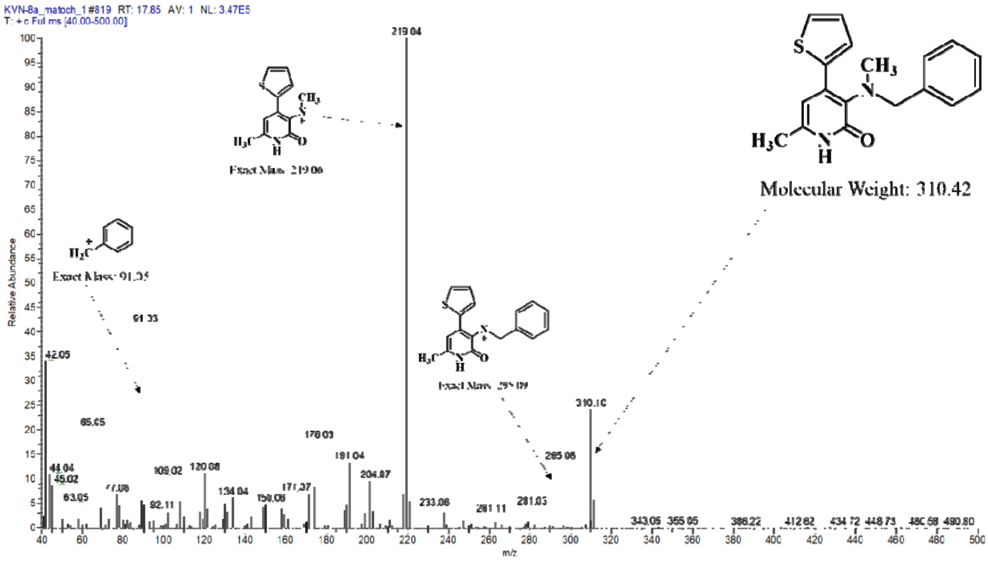

Figure S18. Mass spectrum of 3-(benzyl(methyl)amino)-6-methyl-4-(thiophen-2-yl)pyridin-2(1*H*)–one **9c**

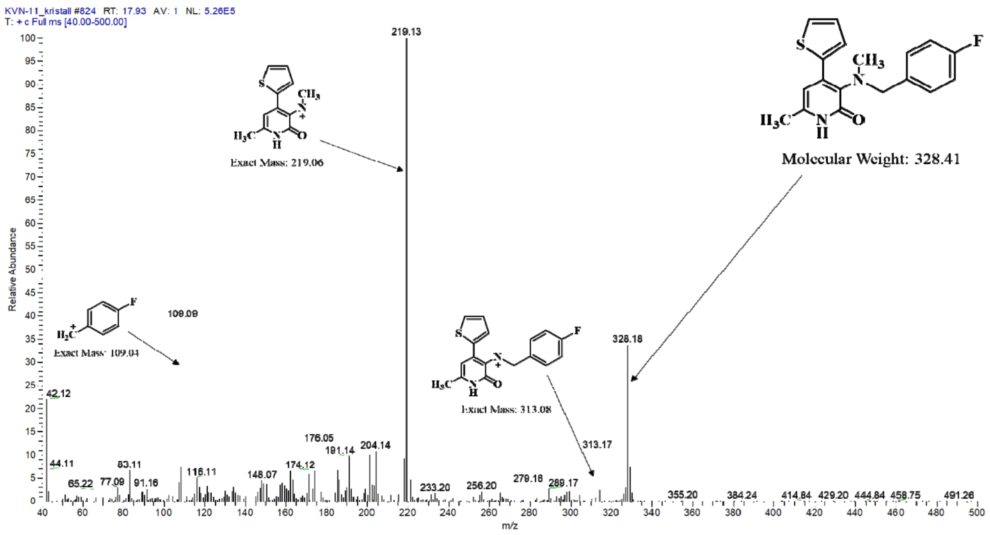

Figure S19. Mass spectrum of 3-((4-fluorobenzyl)(methyl)amino)-6-methyl-4-(thiophen-2-yl)pyridin-2(1*H*)-one **9d**

X-Ray Structural Study of Product

X-ray diffraction data for compounds **6**, **8a**, **9a** were obtained at room temperature with a Bruker Kappa Apex II CCD diffractometer (Bruker, Germany) with Mo-Kα radiation (λ = 0.71073 Å) and a graphite monochromator using ϕ, ω scans of narrow frames. Experimental data reduction was performed using APEX2 v2012.2-0 suite. Absorption corrections were applied empirically using the SADABS-2008/1 programs. X-ray diffraction data for compound **8c** were obtained at room temperature with a Xcalibur Ruby CCD diffractometer (Oxford, United Kingdom) with Cu-Kα radiation (λ = 1.54184 Å) and a graphite monochromator using ω scans. Experimental data reduction and absorption corrections were performed using CrysAlis171 suite. The structures were solved by direct methods and refined by the full-matrix least-squares method against all F2 in the anisotropic approximation using the SHELX 2018 set of programs. The positions of HN hydrogen atoms were located from the difference map and refined isotropically. Positions of the rest of the H atoms were refined with the riding model. CCDC 2467189-2467192 contain the supplementary crystallographic data for this paper. These data can be obtained free of charge from The Cambridge Crystallographic Data Center https://www.ccdc.cam.ac.uk/structures/ (deposited on 25 June 2025).

X-Ray Structural Study of Product

Crystal data **6**: $C_{33}H_{28}N_2O_4$, M = 516.57, monoclinic, space group *C* 2/ *c*, at 296 K: *a* = 17.4826(8), *b* = 13.5157(7), *c* = 24.1512(9) Å, β = 97.384(2)°, *V* = 5659.4(4) Å^3, *Z* = 8, d_{calc} = 1.213 g·cm^{-3}, μ = 0.080 mm^{-1}, a total of 49,730 (θ_{max} = 26.05°), 5593 unique (R_{int} = 0.0533), 3758 [$I > 2\sigma(I)$], 357 parameters. GooF = 0.960, R_1 = 0.0518, wR_2 = 0.1406 [$I > 2\sigma(I)$], R_1 = 0.0857, wR_2 = 0.1740 (all data), max/min diff. peak 0.235/-0.178 e · Å^{-3}. CCDC 2467189.

Crystal data **8a**: $C_{20}H_{18}N_2O_2$, M = 318.36, triclinic, space group *P* −1, at 296 K: *a* = 7.7481(3), *b* = 8.9662(4), *c* = 13.3817(6) Å, *α* = 98.494(2), β = 04.602(2), γ = 103.312(2)°, *V* = 854.29(6) Å^3, *Z* = 2, d_{calc} = 1.238 g·cm^{-3}, μ = 0.081 mm^{-1}, a total of 29182 (θ_{max} = 30.09°), 5007 unique (R_{int} = 0.0398), 3344 [$I > 2\sigma(I)$], 222 parameters. GooF = 1.016, R_1 = 0.0528, wR_2 = 0.1391 [$I > 2\sigma(I)$], R_1 = 0.0867, wR_2 = 0.1709 (all data), max/min diff. peak 0.268/−0.192 e · Å^{-3}. CCDC 2467190.

Crystal data **8c**: $C_{20}H_{17}FN_2O_2$, M = 336.35, triclinic, space group *P* −1, at 296 K: *a* = 7.8797(8), *b* = 8.9057(10), *c* = 13.405(2) Å, *α* = 97.170(11), β = 106.466(11), γ = 103.214(9)°, *V* = 859.9(2) Å^3, *Z* = 2, d_{calc} = 1.299 g·cm^{-3}, μ = 0.758 mm^{-1}, a total of 5604 (θ_{max} = 75.99°), 3430 unique (R_{int} = 0.0602), 1479 [$I > 2\sigma(I)$], 231

parameters. GooF = 0.932, R_1 = 0.0600, wR_2 = 0.1090 [$I > 2\sigma(I)$], R_1 = 0.1503, wR_2 = 0.1493 (all data), max/min diff. peak 0.165/–0.217 e Å^{-3}. CCDC 2467191.

Crystal data **9a**: $C_{20}H_{20}N_2O$, M = 304.38, monoclinic, space group $P\,2_1/n$, at 296 K: a = 10.7039(4), b = 7.8392(3), c = 21.0351(8) Å, β = 102.114(2)°, V = 1725.75(11) Å^3, Z = 4, d_{calc} = 1.172 g·cm^{-3}, μ = 0.073 mm^{-1}, a total of 31,334 (θ_{max} = 27.94°), 4137 unique (R_{int} = 0.0508), 3160 [$I > 2\sigma(I)$], 214 parameters. GooF = 1.006, R_1 = 0.0555, wR_2 = 0.1473 [$I > 2\sigma(I)$], R_1 = 0.0757, wR_2 = 0.1736 (all data), max/min diff. peak 0.214/–0.206 e · Å^{-3}. CCDC 2467192.

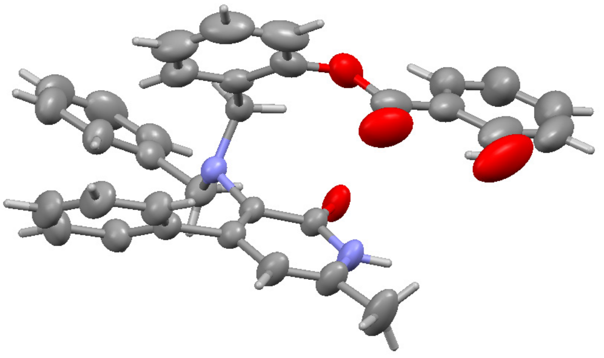

6

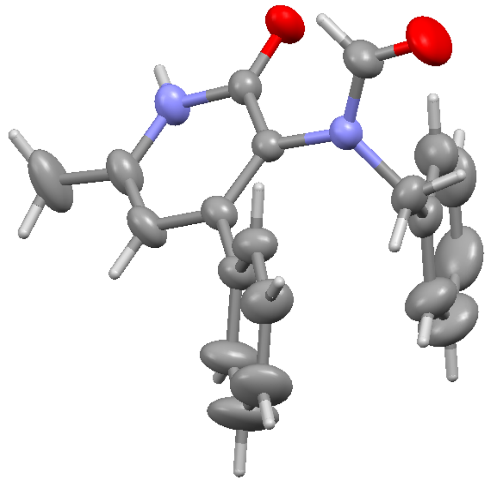

8a

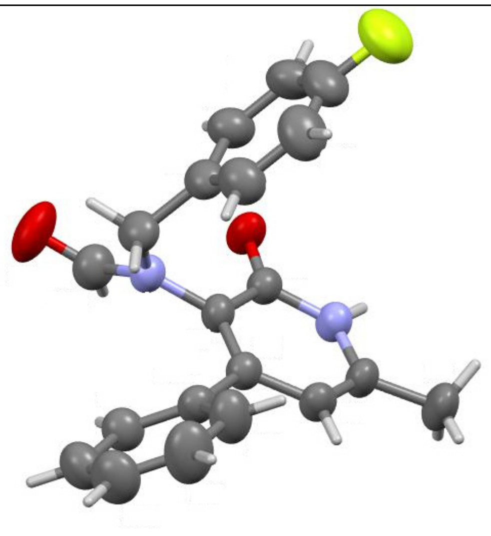

8c

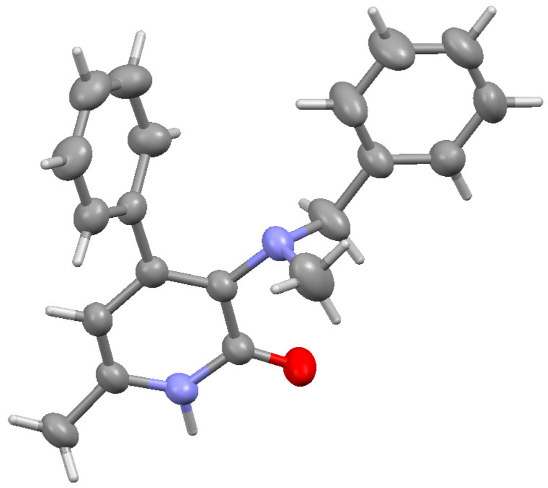

9a

Figure S20. X-ray crystal structure of compounds **6** (CCDC 2467189); **8a** (CCDC 2467190; **8c** (CCDC 2467191); **9** (CCDC 2467192)
